# Supplementary figures and images for: Control of meiotic entry by dual inhibition of a key mitotic transcription factor
Source: eLife. 2024 Feb 27;12:RP90425. doi: 10.7554/eLife.90425 (PMC10939502; doi:10.7554/eLife.90425)

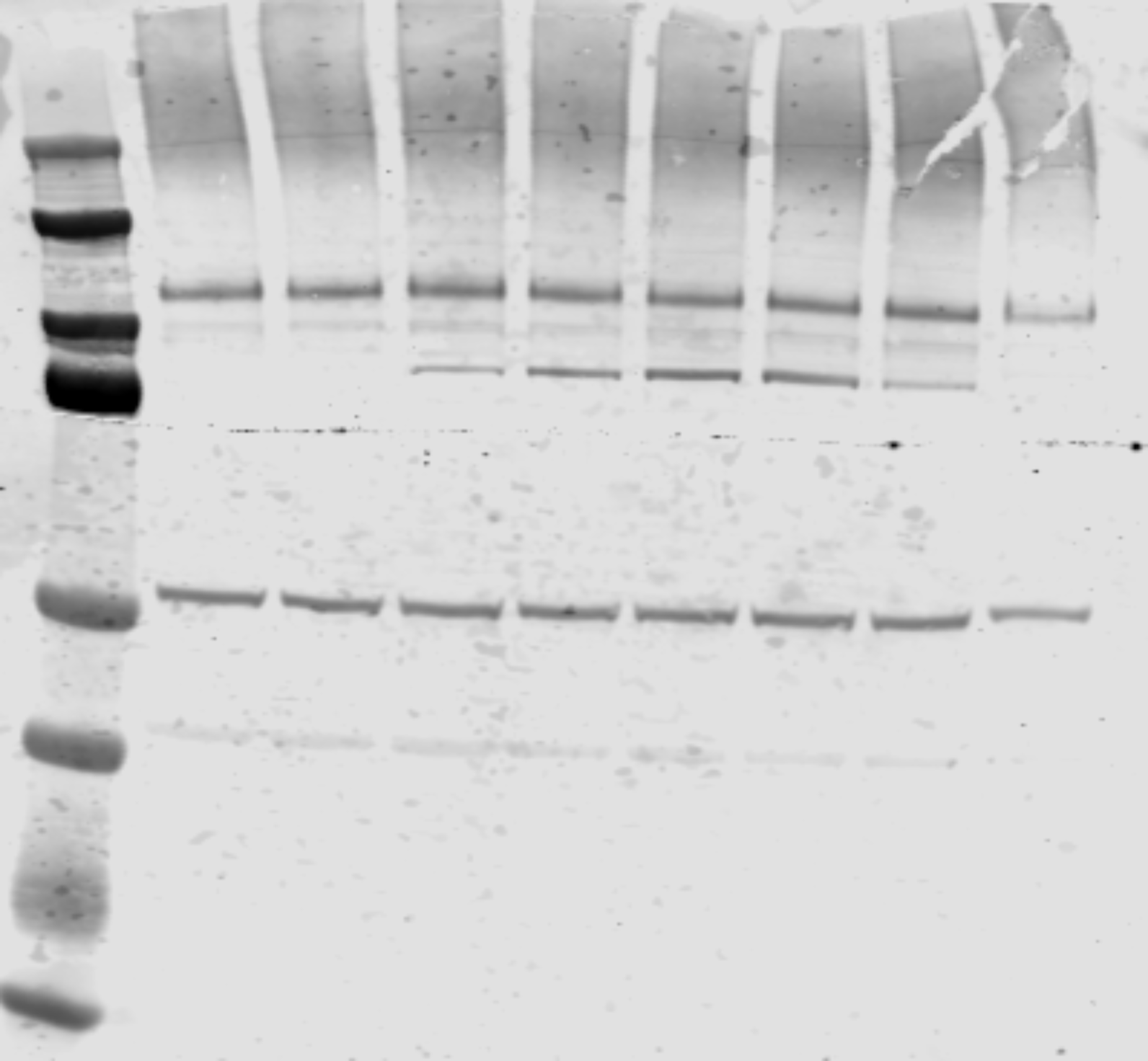

Supplement: Figure 1—source data 1. [file elife-90425-fig1-data1.zip › Figure 1 source data 1/1B_Mbp1_Hxk2_uncropped.tif]

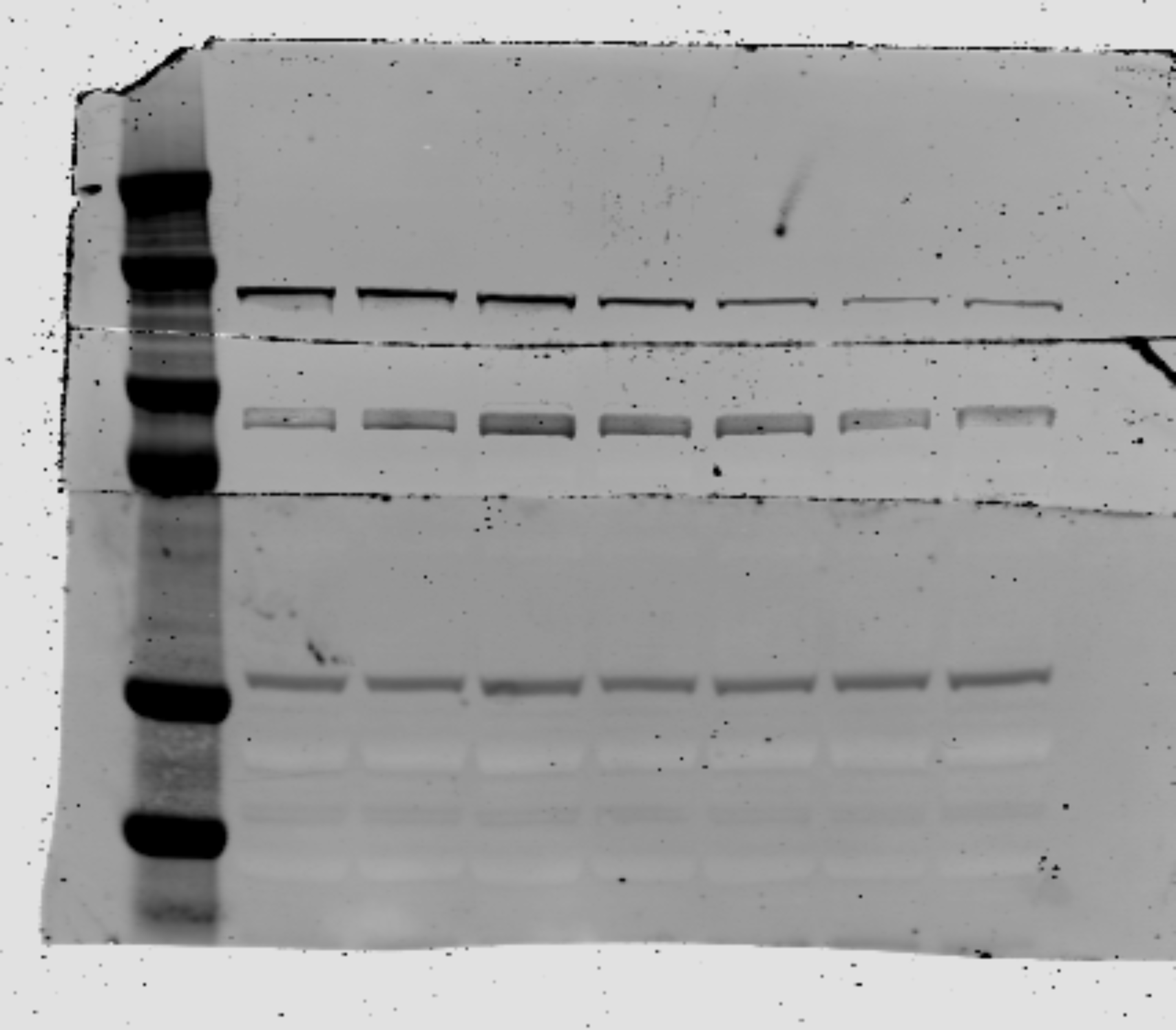

Supplement: Figure 1—source data 1. [file elife-90425-fig1-data1.zip › Figure 1 source data 1/1B_Swi4_Swi6_Hxk2_uncropped.tif]

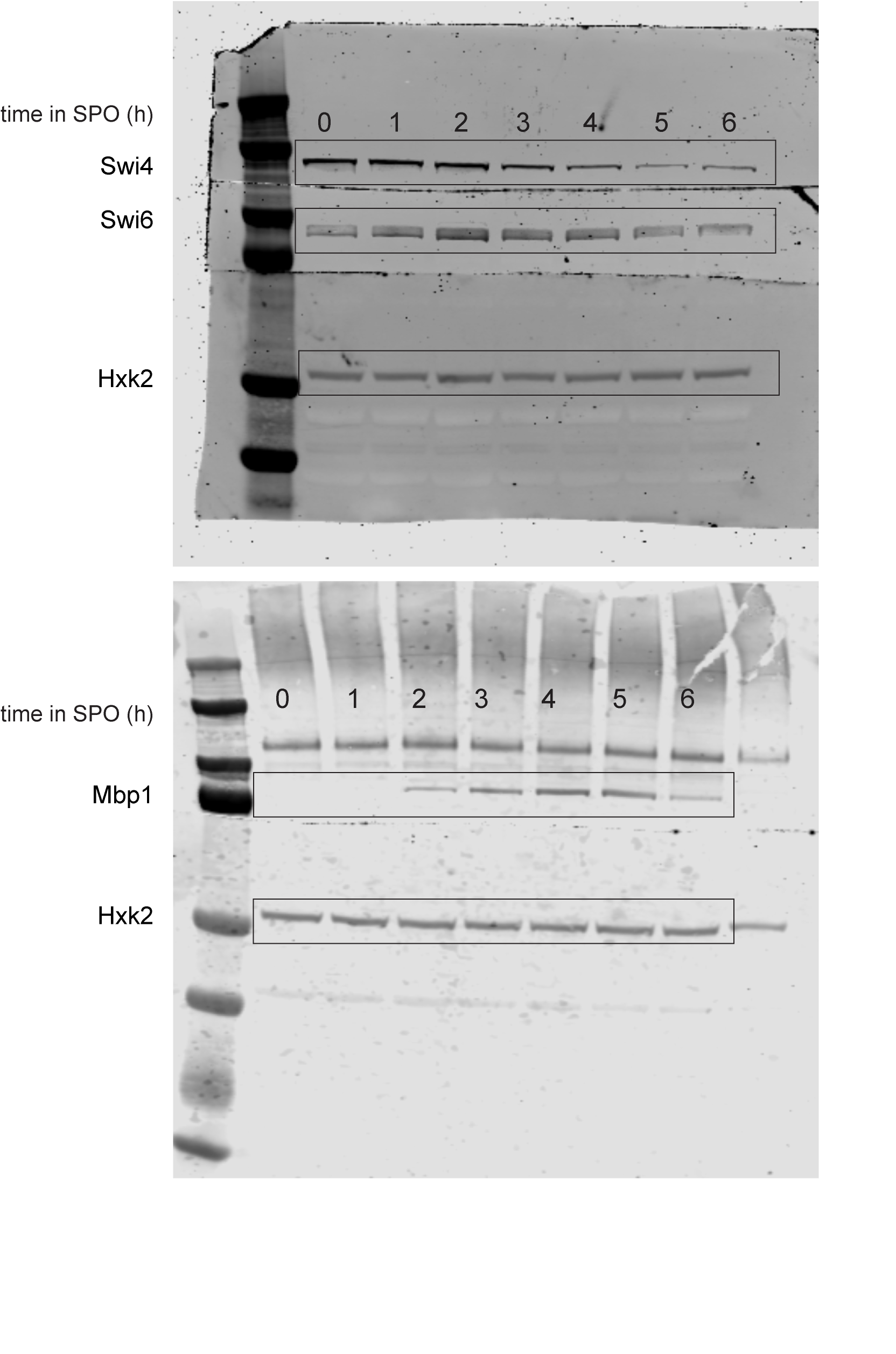

Supplement: Figure 1—source data 2. [file elife-90425-fig1-data2.zip › Figure 1 source data 2/1B_uncropped_with-labels.tif]

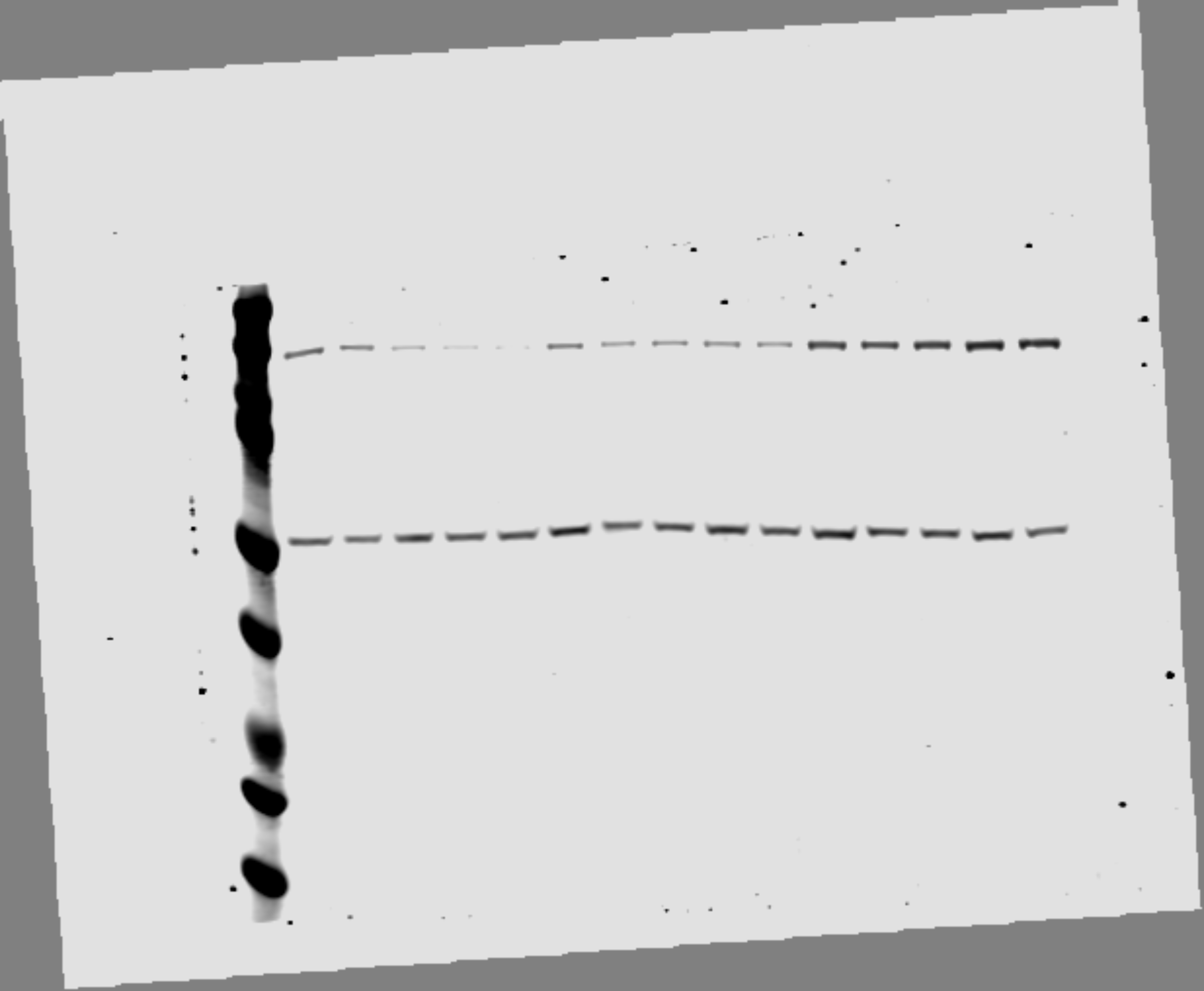

Supplement: Figure 1—figure supplement 1—source data 1. [file elife-90425-fig1-figsupp1-data1.zip › Figure 1-figure supplement 1 source data 1/Figure 1-figure supplement 1_uncropped.tif]

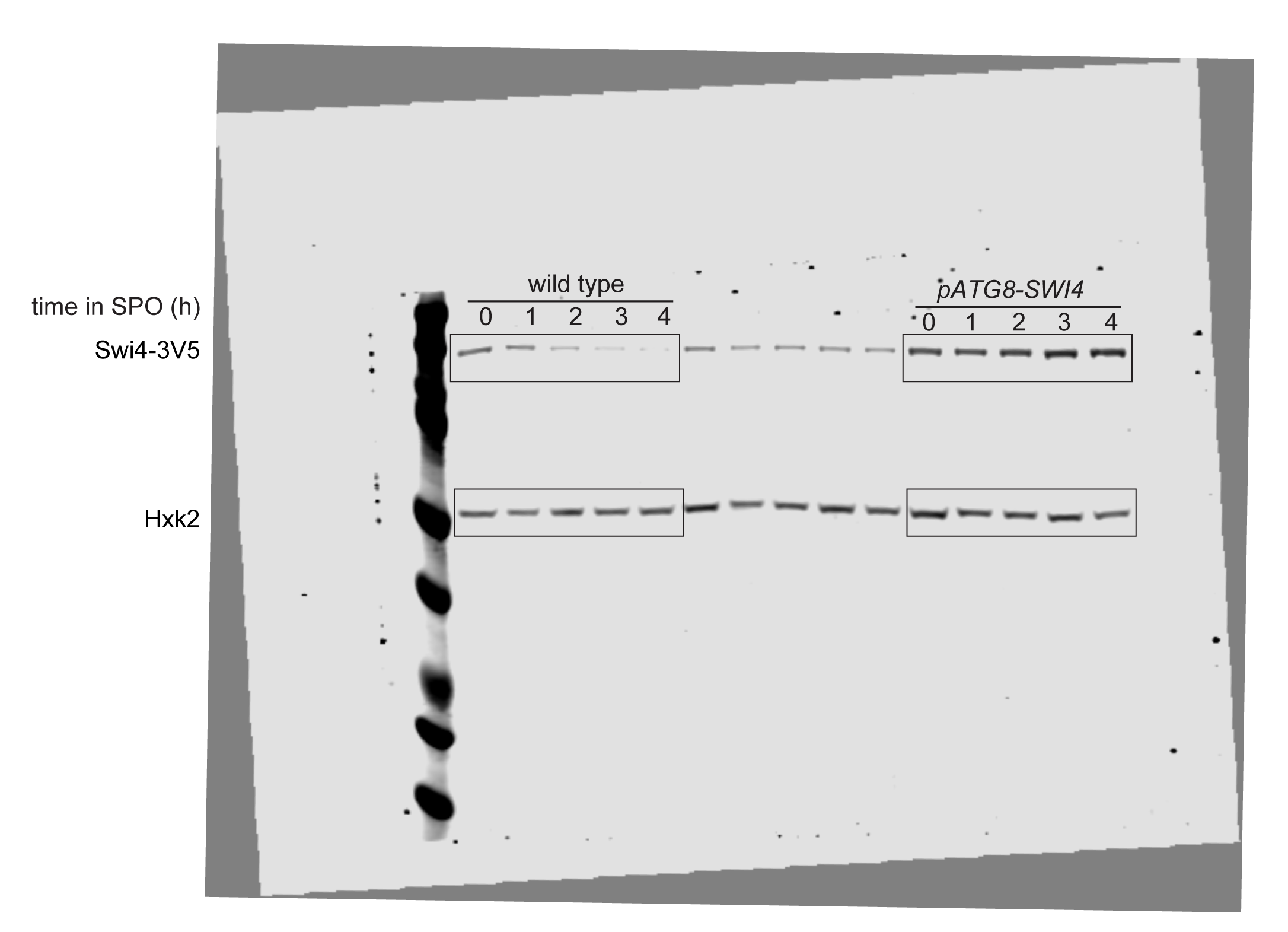

Supplement: Figure 1—figure supplement 1—source data 2. [file elife-90425-fig1-figsupp1-data2.zip › Figure 1-figure supplement 1 source data 2/Figure 1-figure supplement 1_uncropped_with-labels.tif]

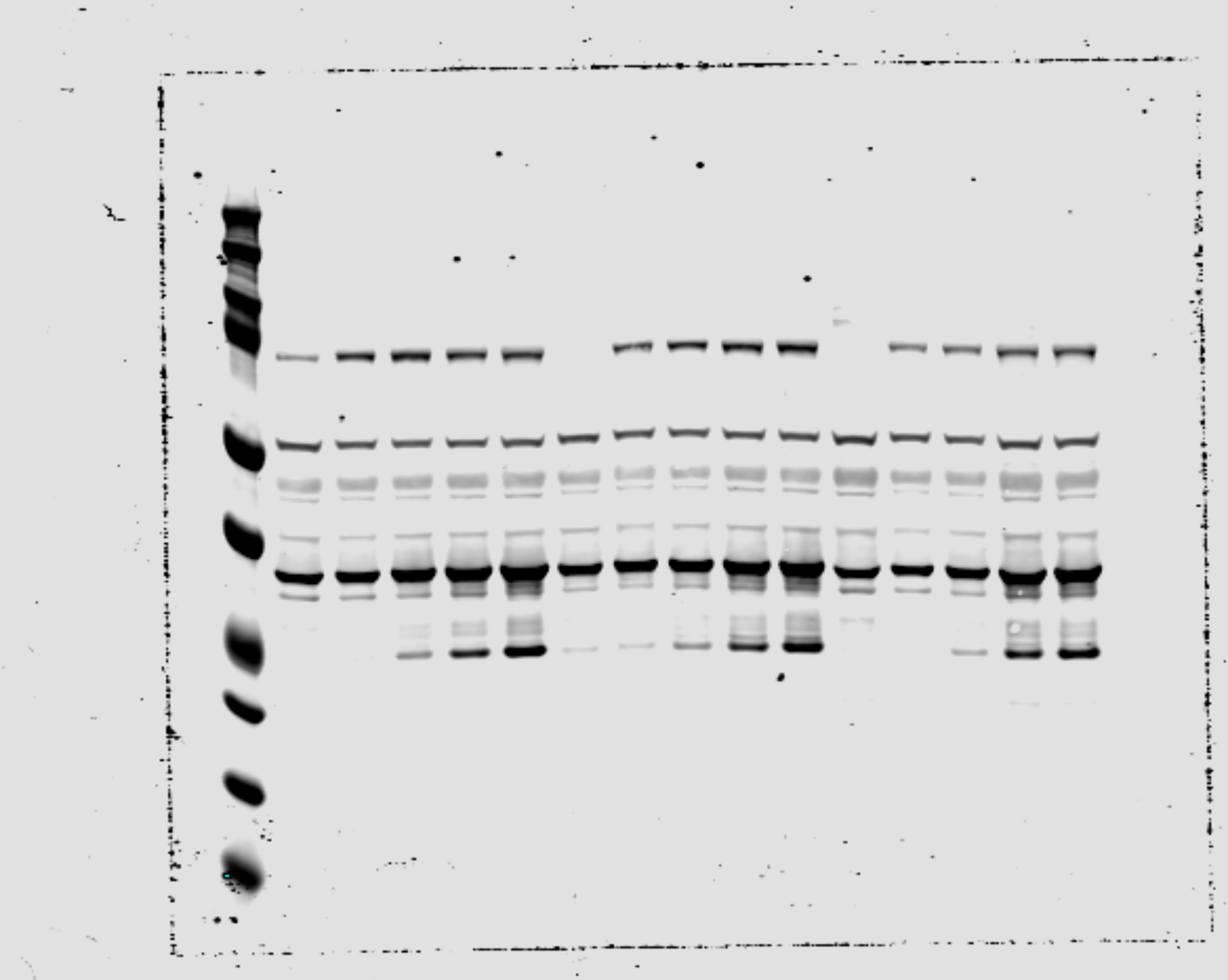

Supplement: Figure 2—figure supplement 1—source data 1. [file elife-90425-fig2-figsupp1-data1.zip › Figure 2-figure supplement 1 source data 1/1C_Ime1_Hxk2.tif]

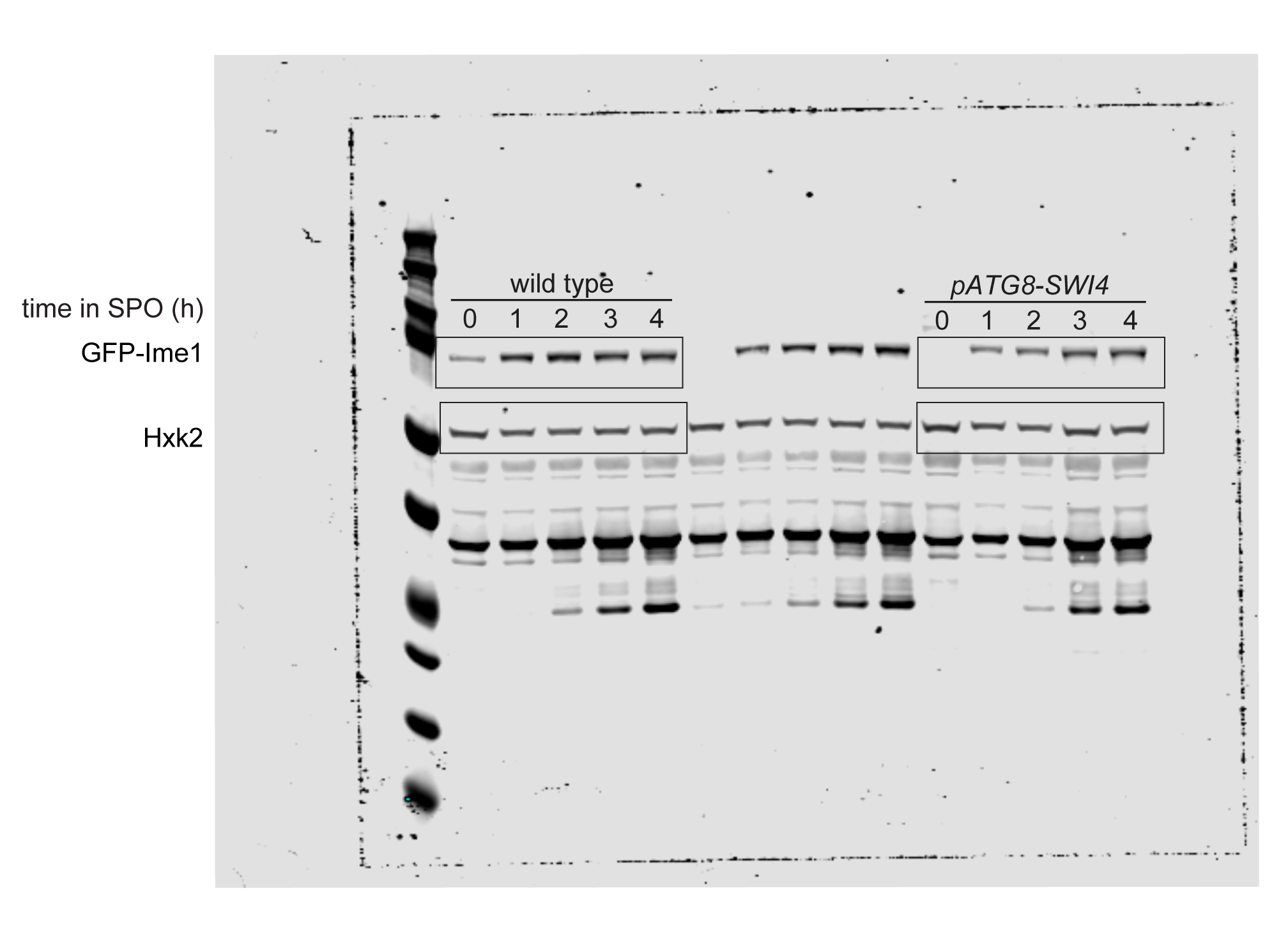

Supplement: Figure 2—figure supplement 1—source data 2. [file elife-90425-fig2-figsupp1-data2.zip › Figure 2-figure supplement 1 source data 2/1C_uncropped_with-labels.tif]

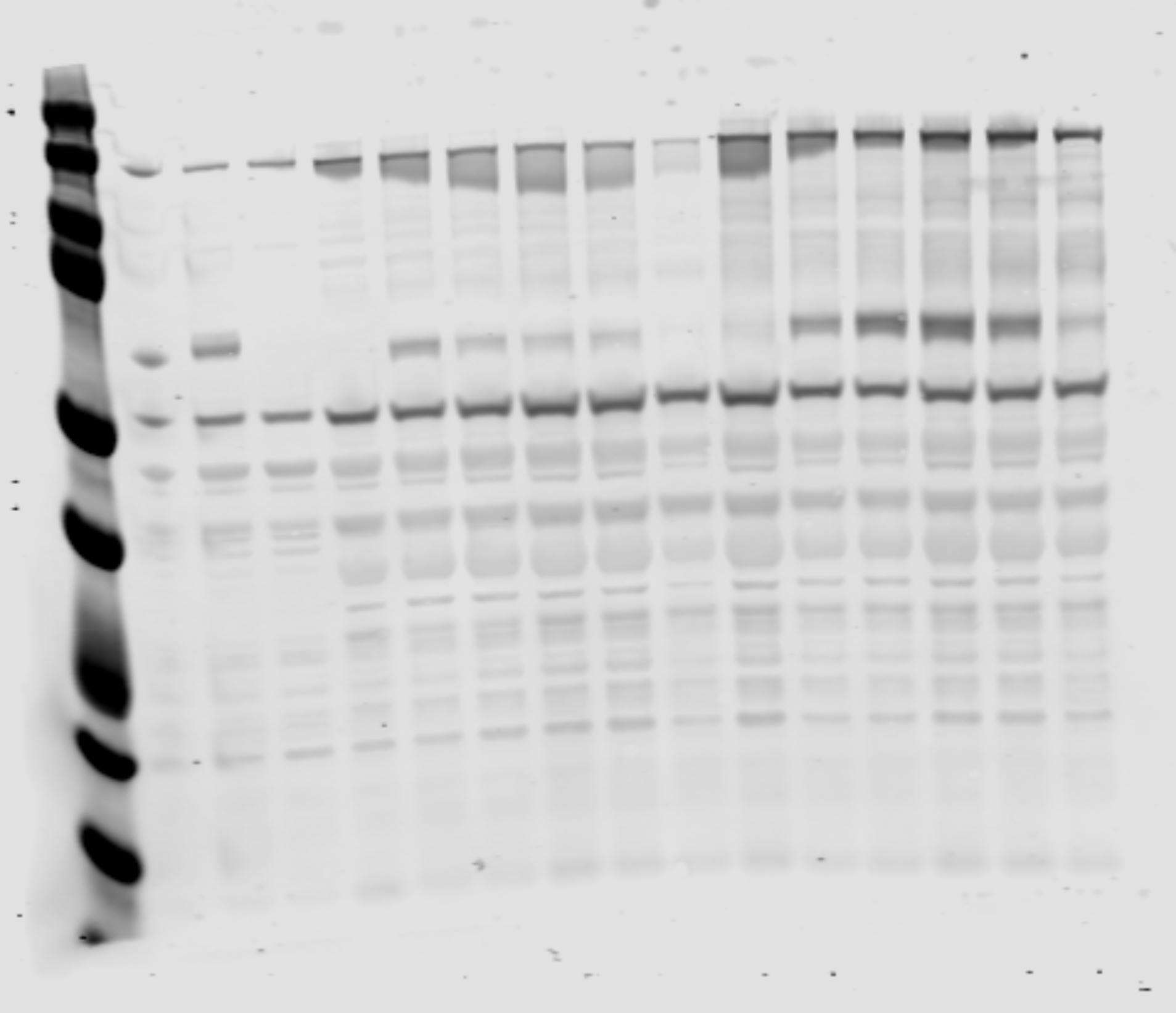

Supplement: Figure 3—source data 1. [file elife-90425-fig3-data1.zip › Figure 3 source data 1/3A_Cln1_Hxk2_uncropped.tif]

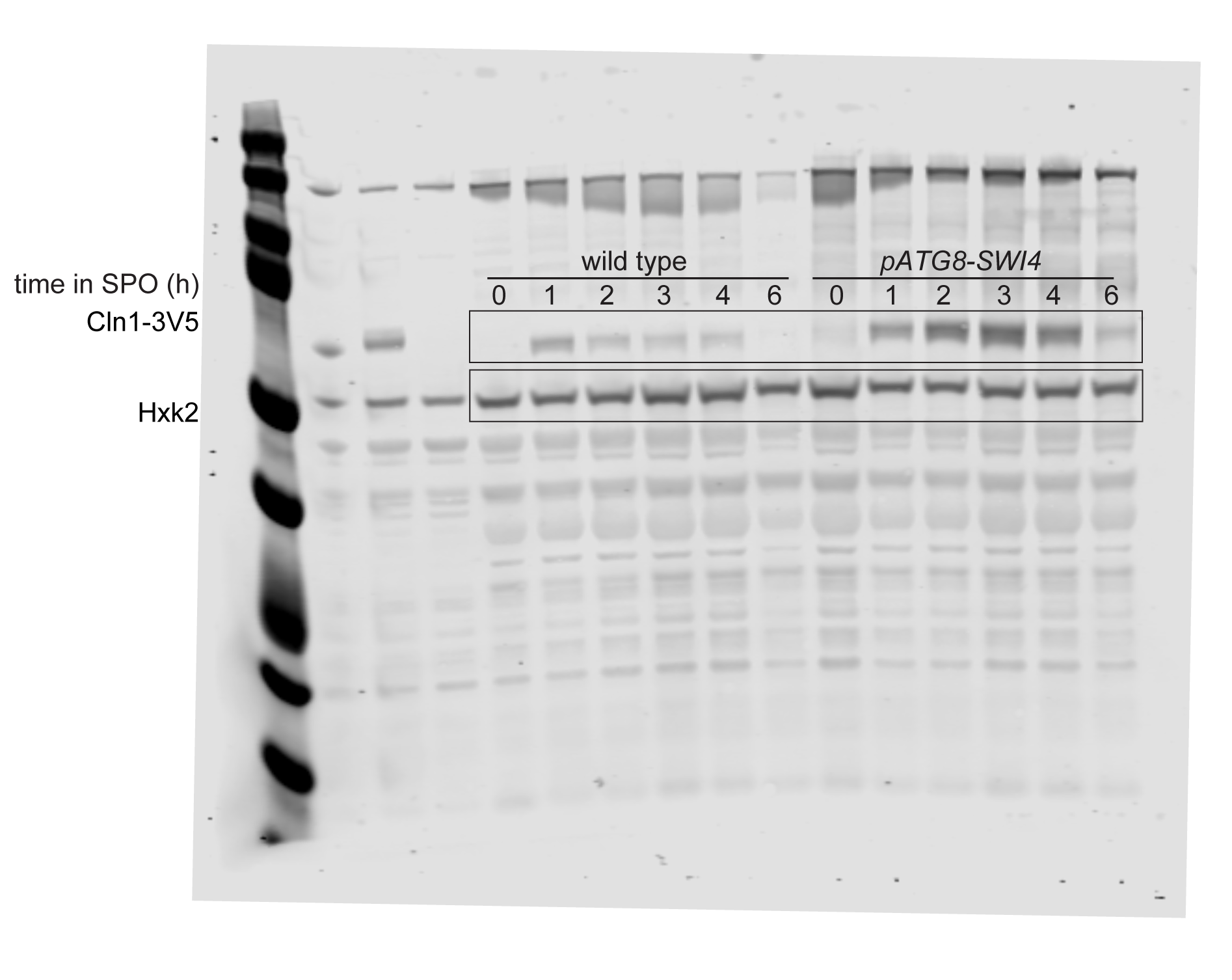

Supplement: Figure 3—source data 2. [file elife-90425-fig3-data2.zip › Figure 3 source data 2/3A_uncropped_with-labels.tif]

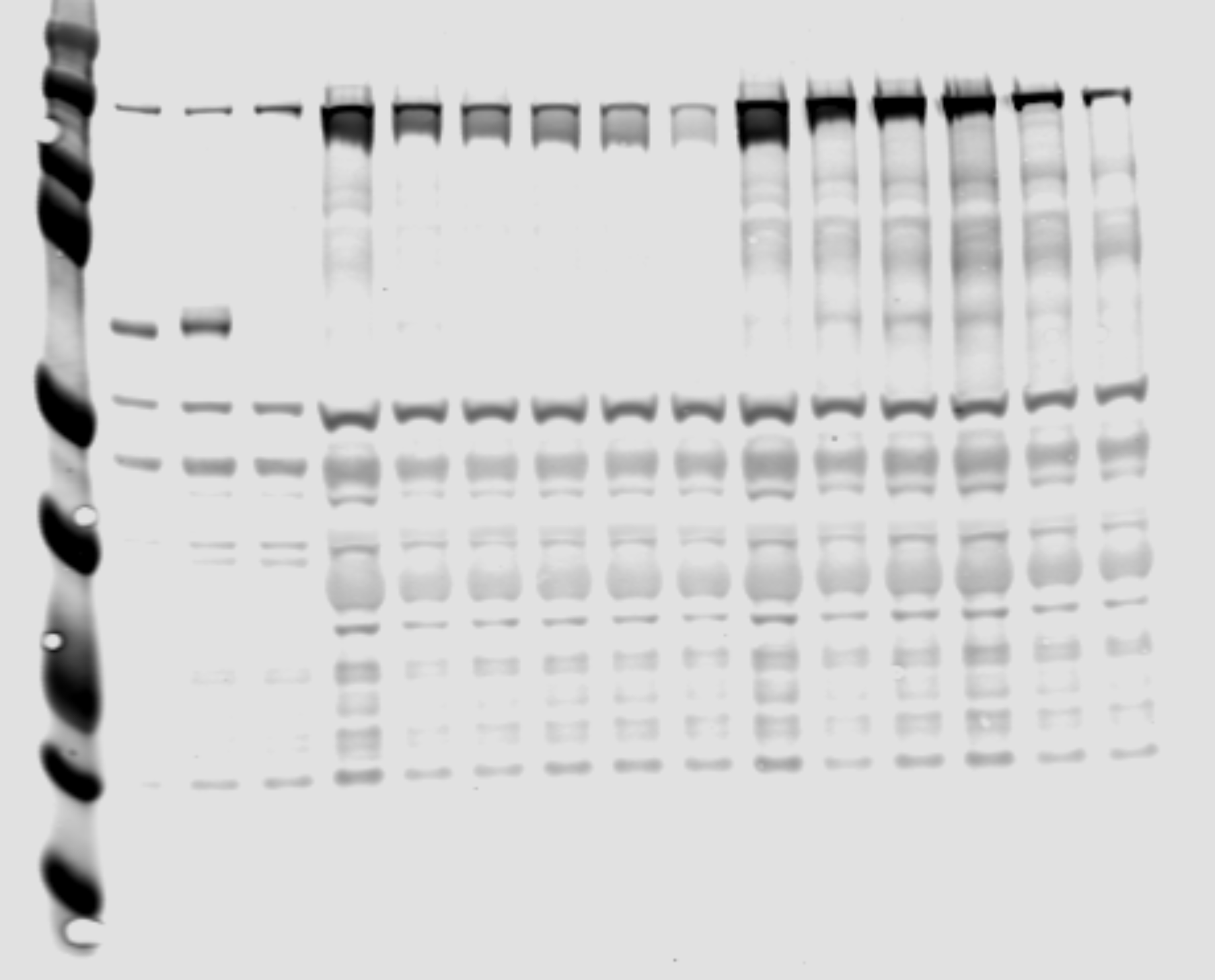

Supplement: Figure 3—source data 3. [file elife-90425-fig3-data3.zip › Figure 3 source data 3/3C_Cln2_Hxk2_uncropped.tif]

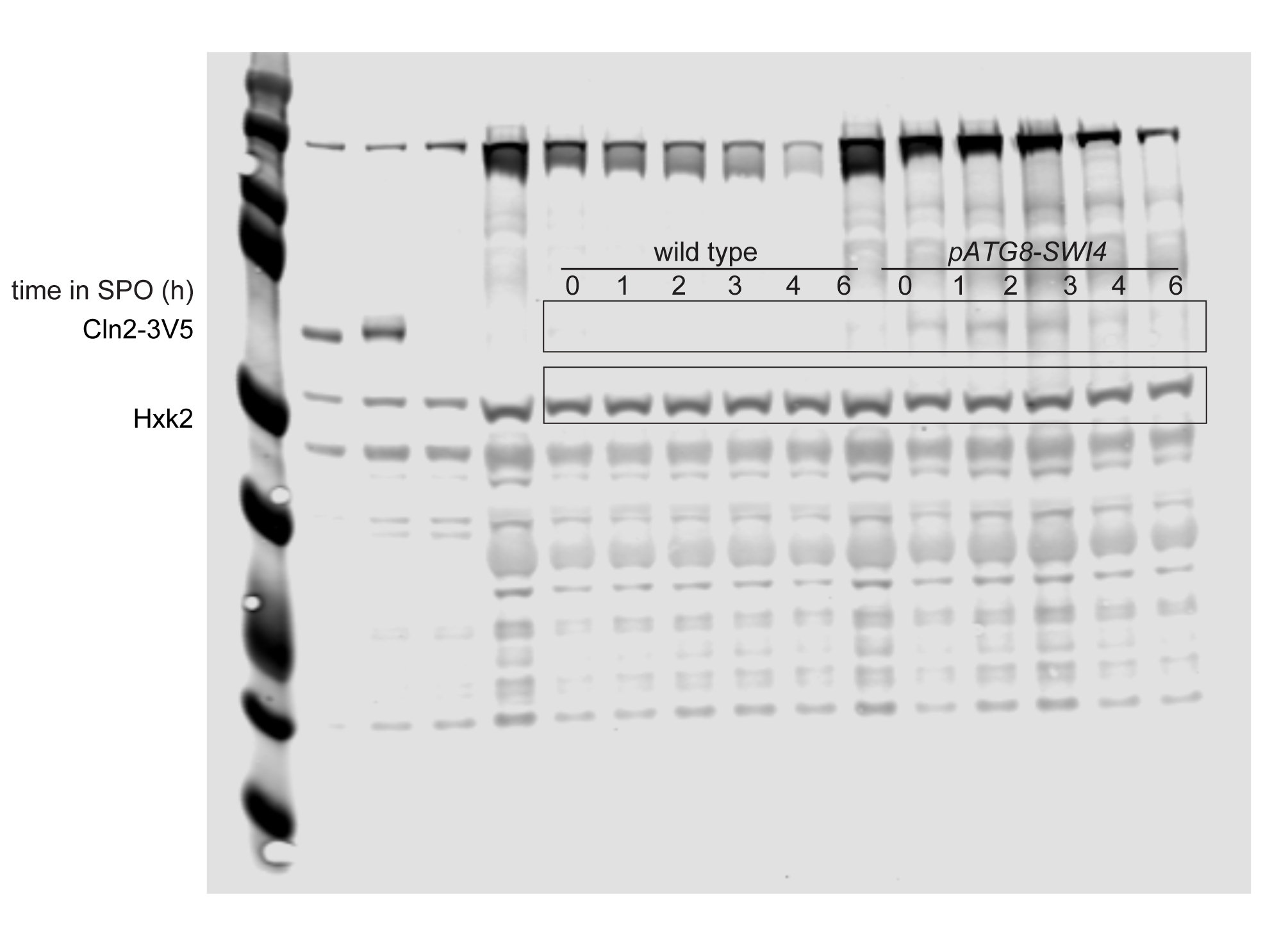

Supplement: Figure 3—source data 4. [file elife-90425-fig3-data4.zip › Figure 3 source data 4/3C_uncropped_with-labels.tif]

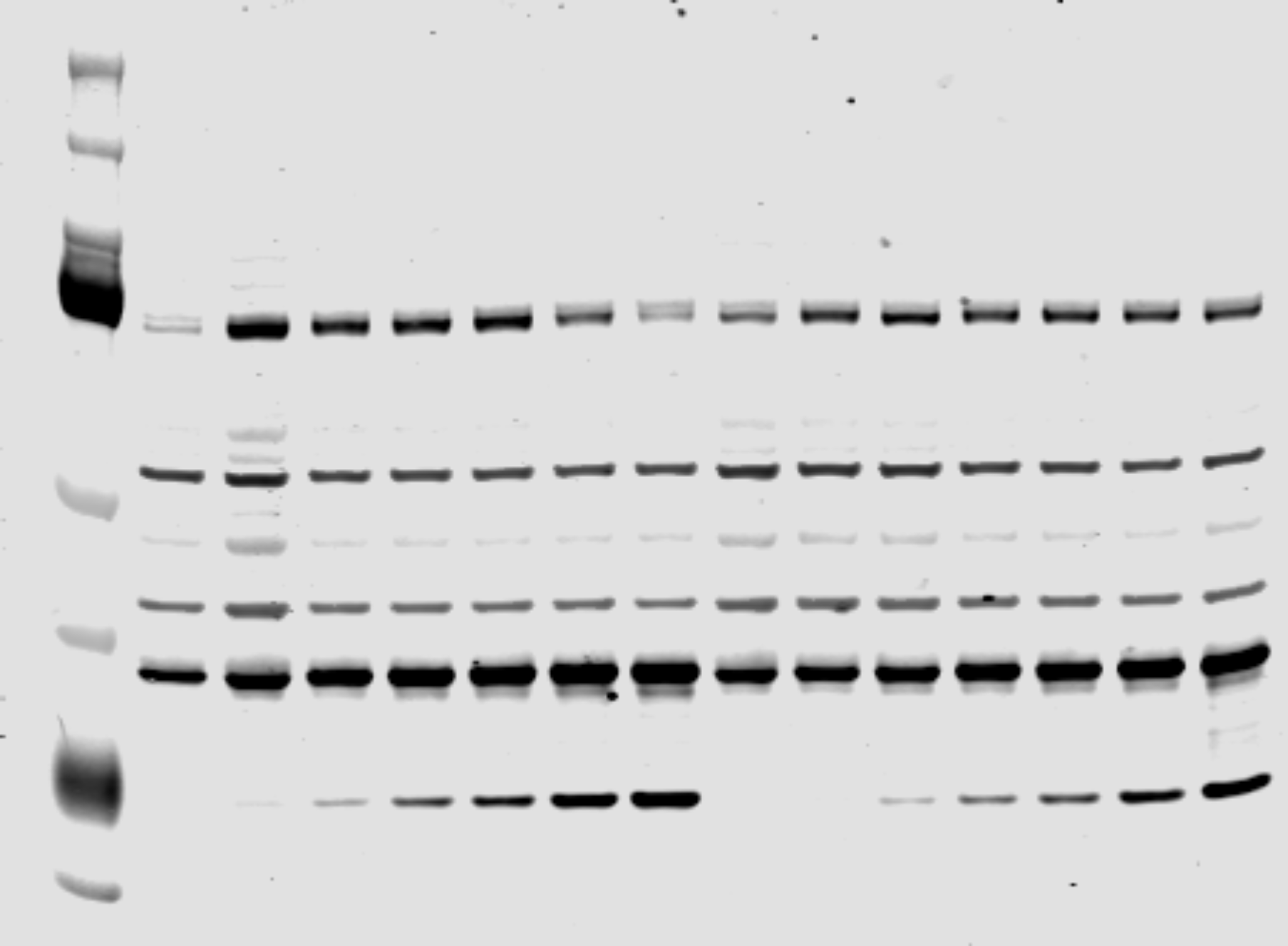

Supplement: Figure 4—figure supplement 2—source data 1. [file elife-90425-fig4-figsupp2-data1.zip › Figure 4-figure supplement 2 source data 1/Figure 4-figure supplement 2_Ime1_Hxk2_uncropped.tif]

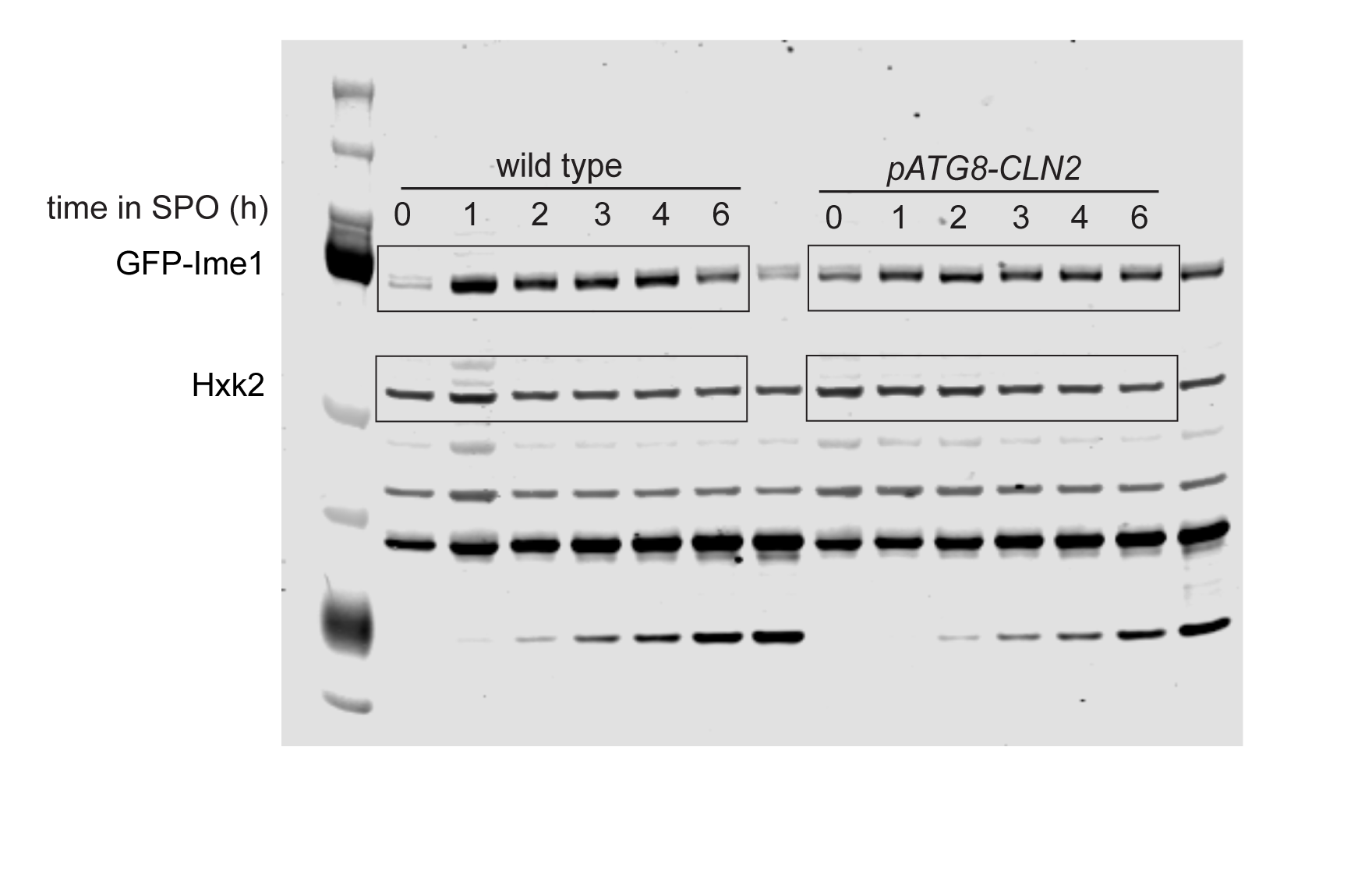

Supplement: Figure 4—figure supplement 2—source data 2. [file elife-90425-fig4-figsupp2-data2.zip › Figure 4-figure supplement 2 source data 2/Figure 4-figure supplement 2_uncropped_with-labels.tif]

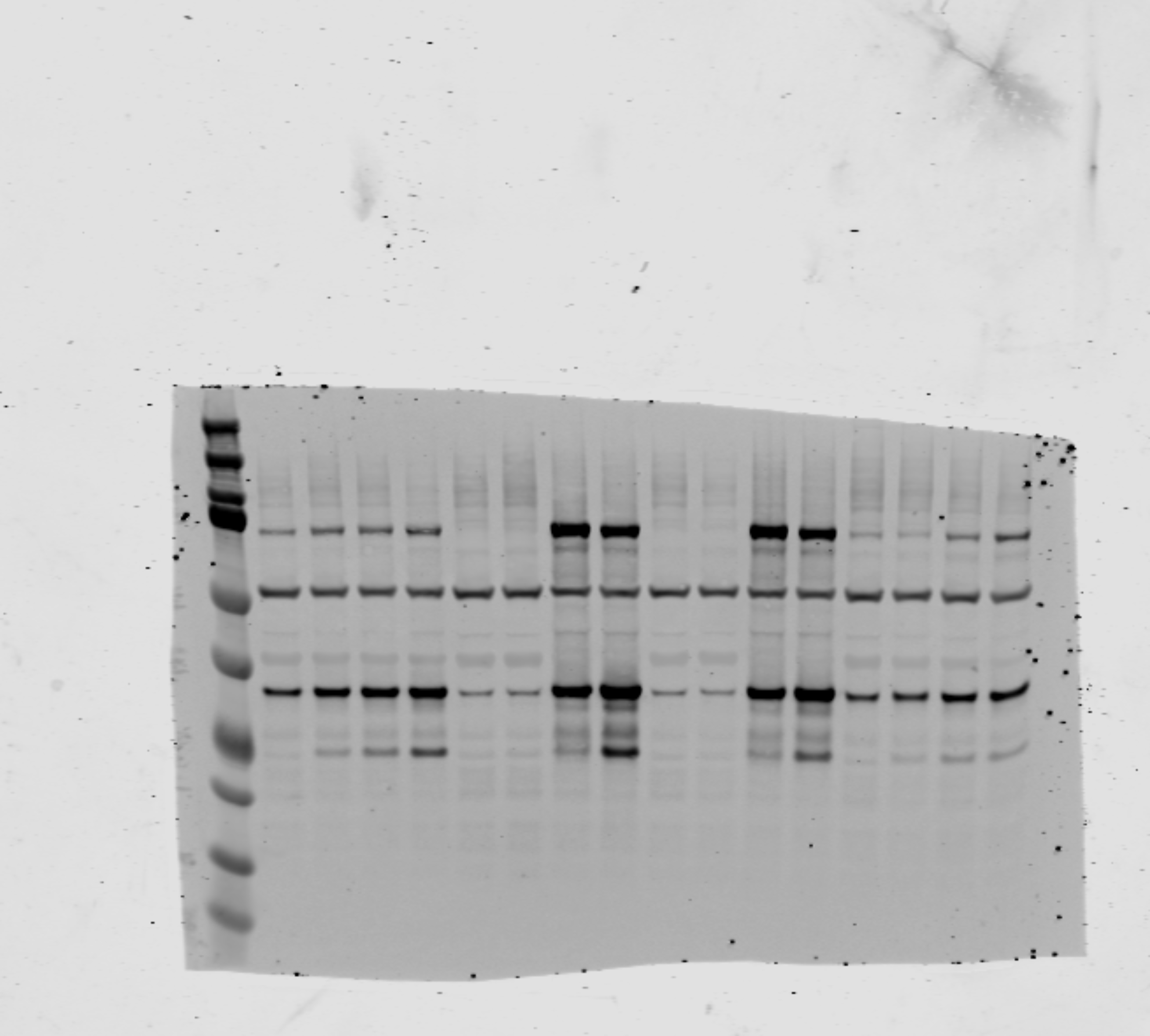

Supplement: Figure 4—figure supplement 4—source data 1. [file elife-90425-fig4-figsupp4-data1.zip › Figure 4-figure supplement 4 source data 1/Figure 4-figure supplement 4_Ime1_Hxk2_uncropped.tif]

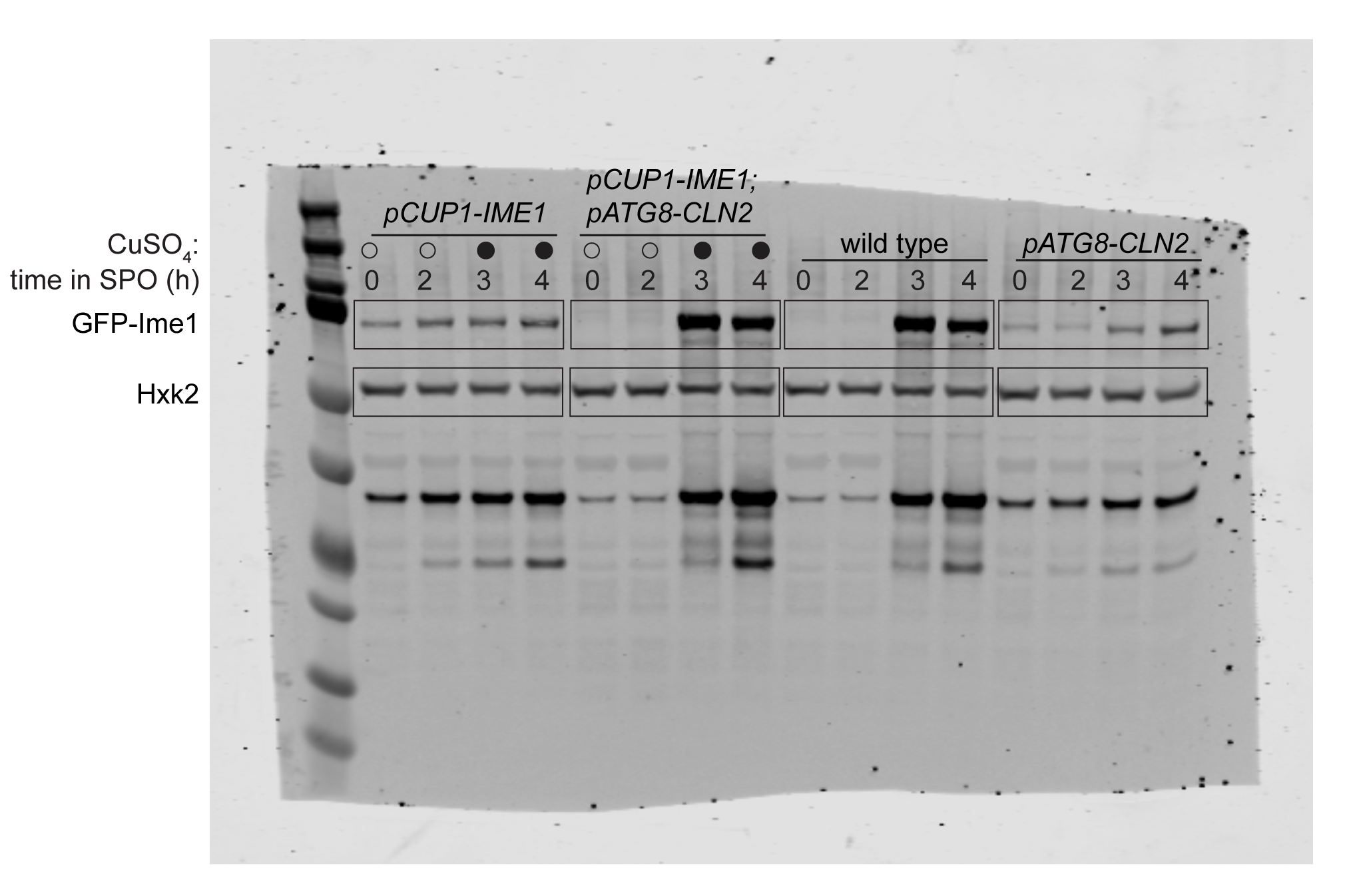

Supplement: Figure 4—figure supplement 4—source data 2. [file elife-90425-fig4-figsupp4-data2.zip › Figure 4-figure supplement 4 source data 2/Figure 4-figure supplement 4_uncroppped_with-labels.tif]

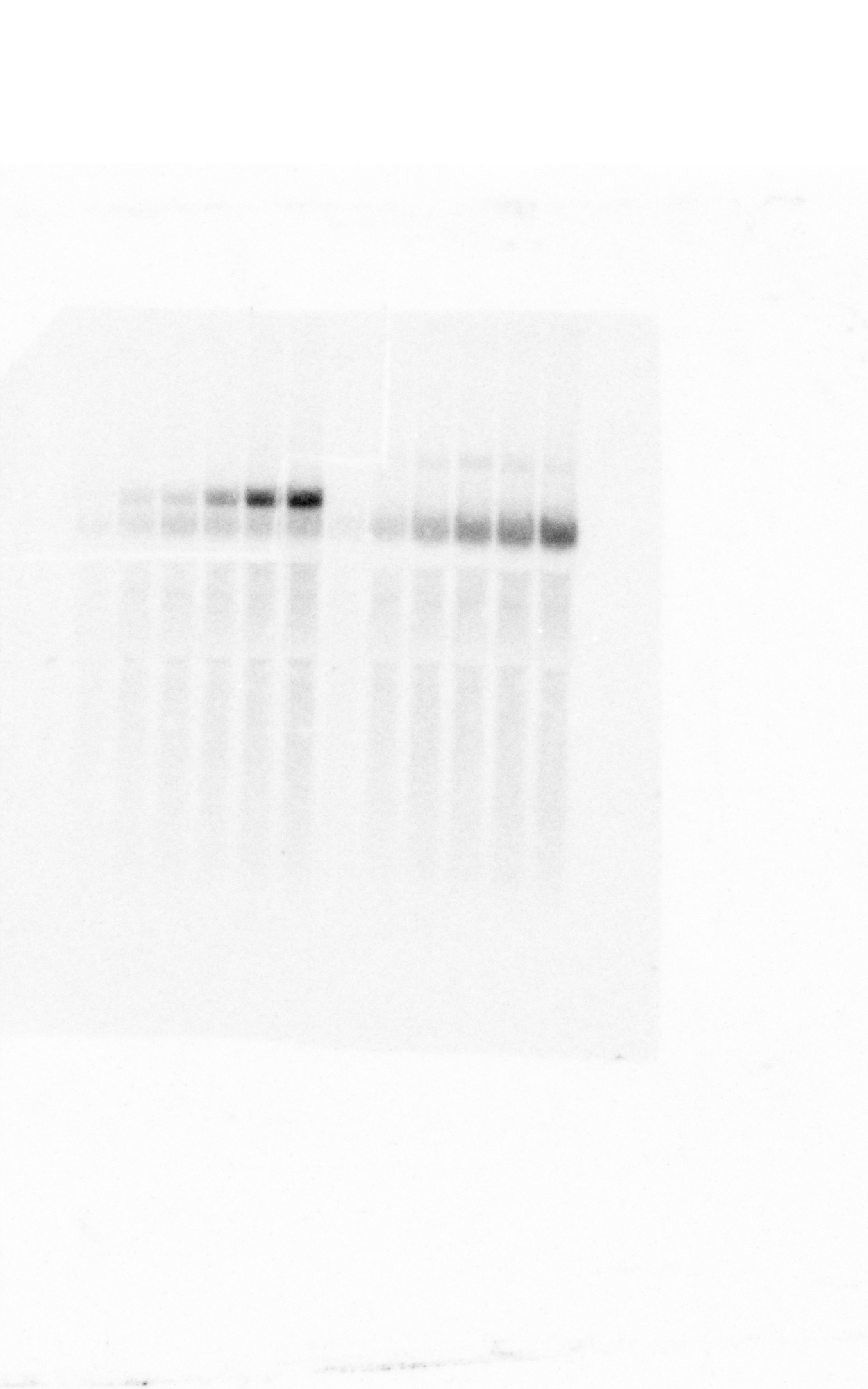

Supplement: Figure 5—source data 1. [file elife-90425-fig5-data1.zip › Figure 5 source data 1/5E_SWI4LUTI_RNA-blot_uncropped.tif]

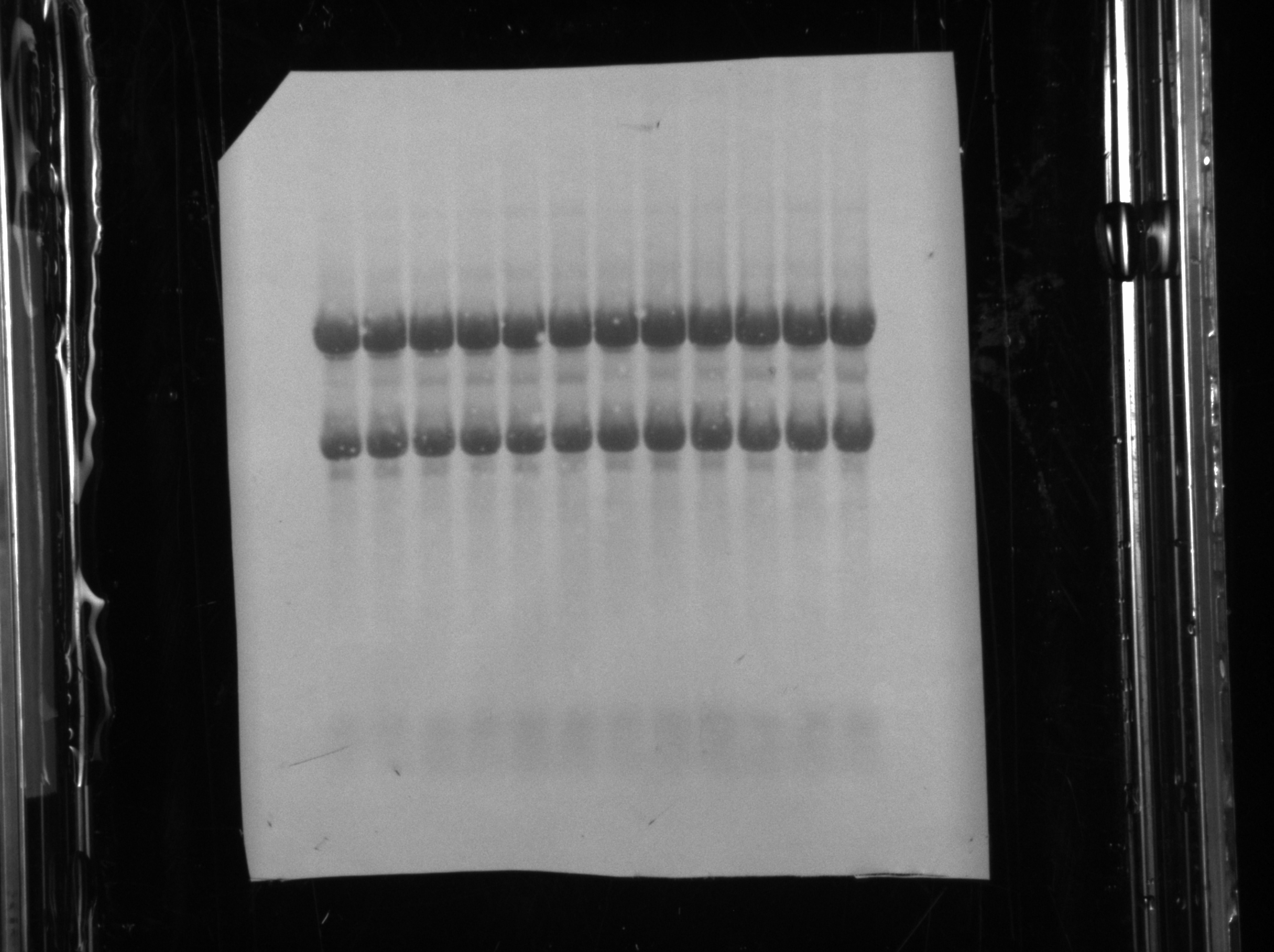

Supplement: Figure 5—source data 2. [file elife-90425-fig5-data2.zip › Figure 5 source data 2/5E_rRNA_methylene-blue_RNA-blot_uncropped.tif]

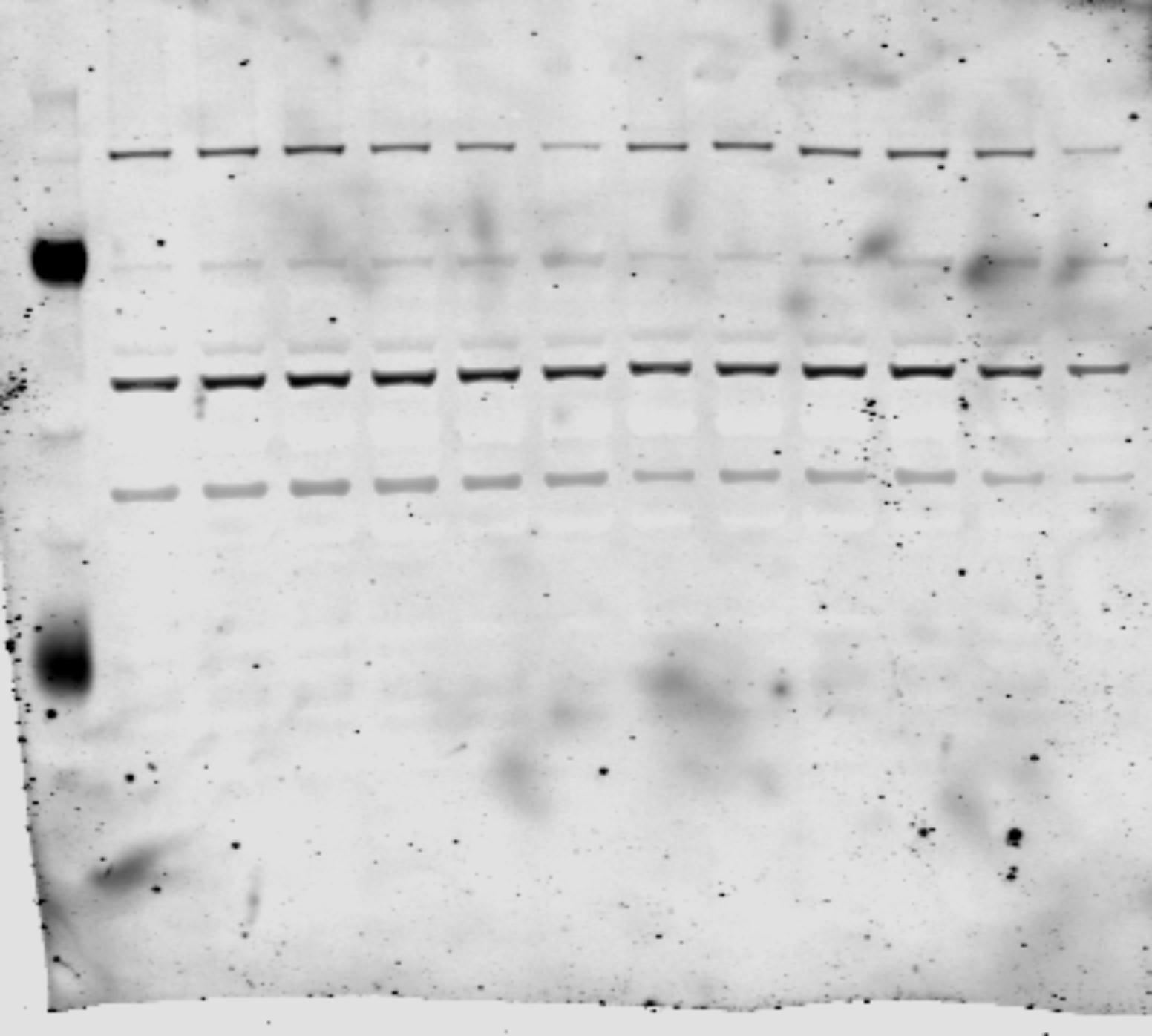

Supplement: Figure 5—source data 3. [file elife-90425-fig5-data3.zip › Figure 5 source data 3/5E_Swi4_Hxk2_Protein-blot_uncropped.tif]

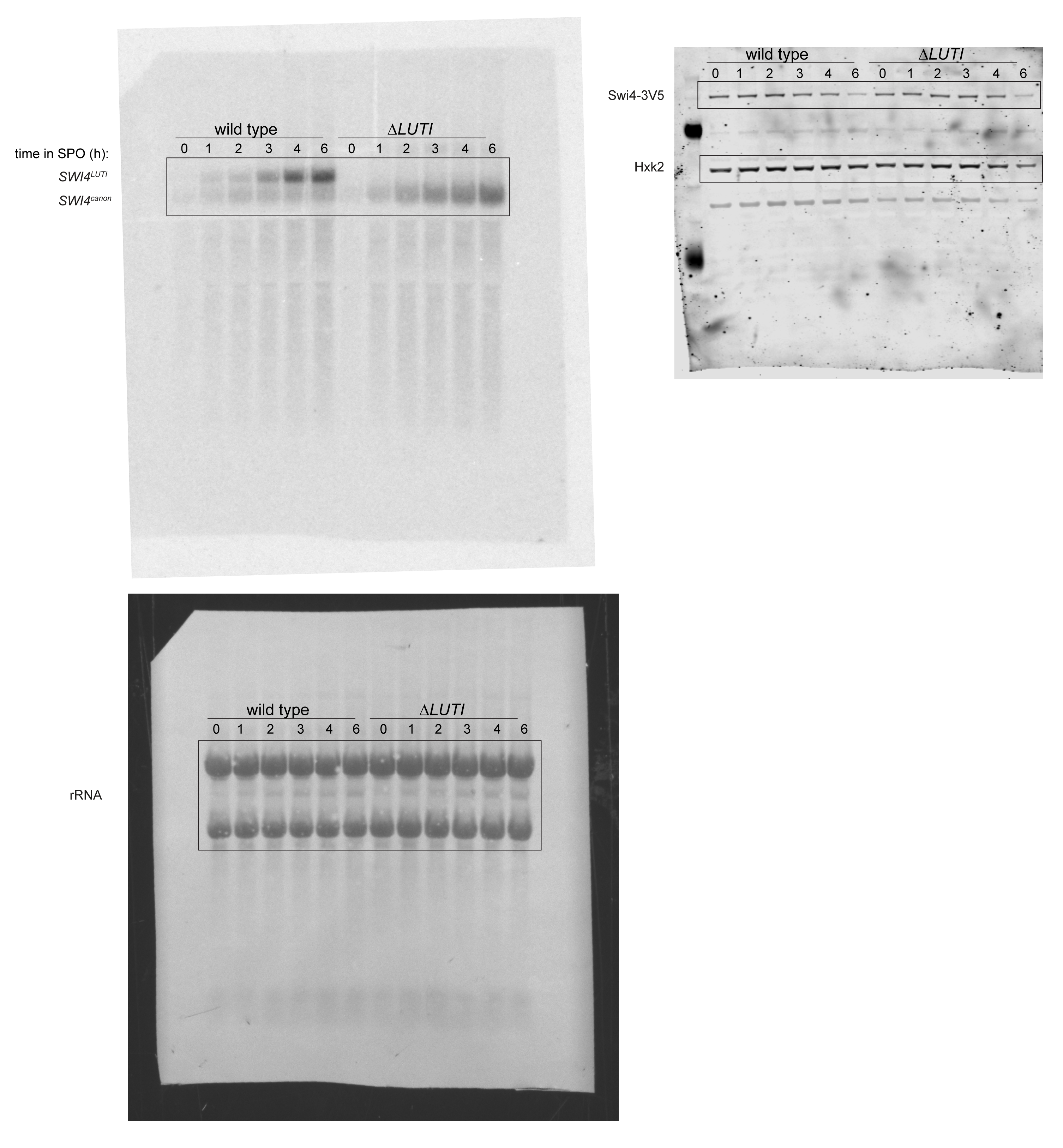

Supplement: Figure 5—source data 4. [file elife-90425-fig5-data4.zip › Figure 5 source data 4/5E_uncropped_with-labels.tif]

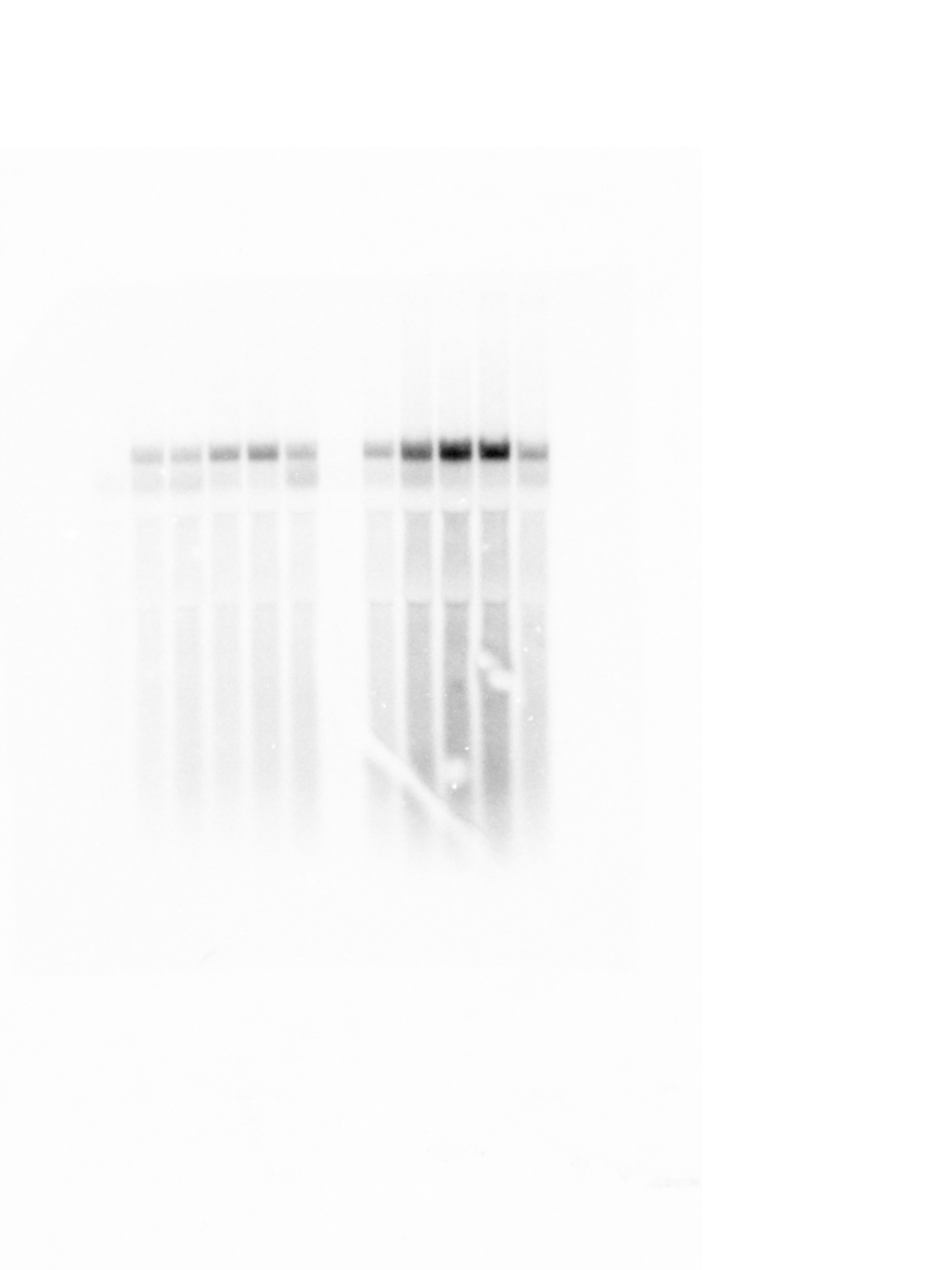

Supplement: Figure 5—source data 5. [file elife-90425-fig5-data5.zip › Figure 5 source data 5/5G_SWI4LUTI_RNA-blot_uncropped.tif]

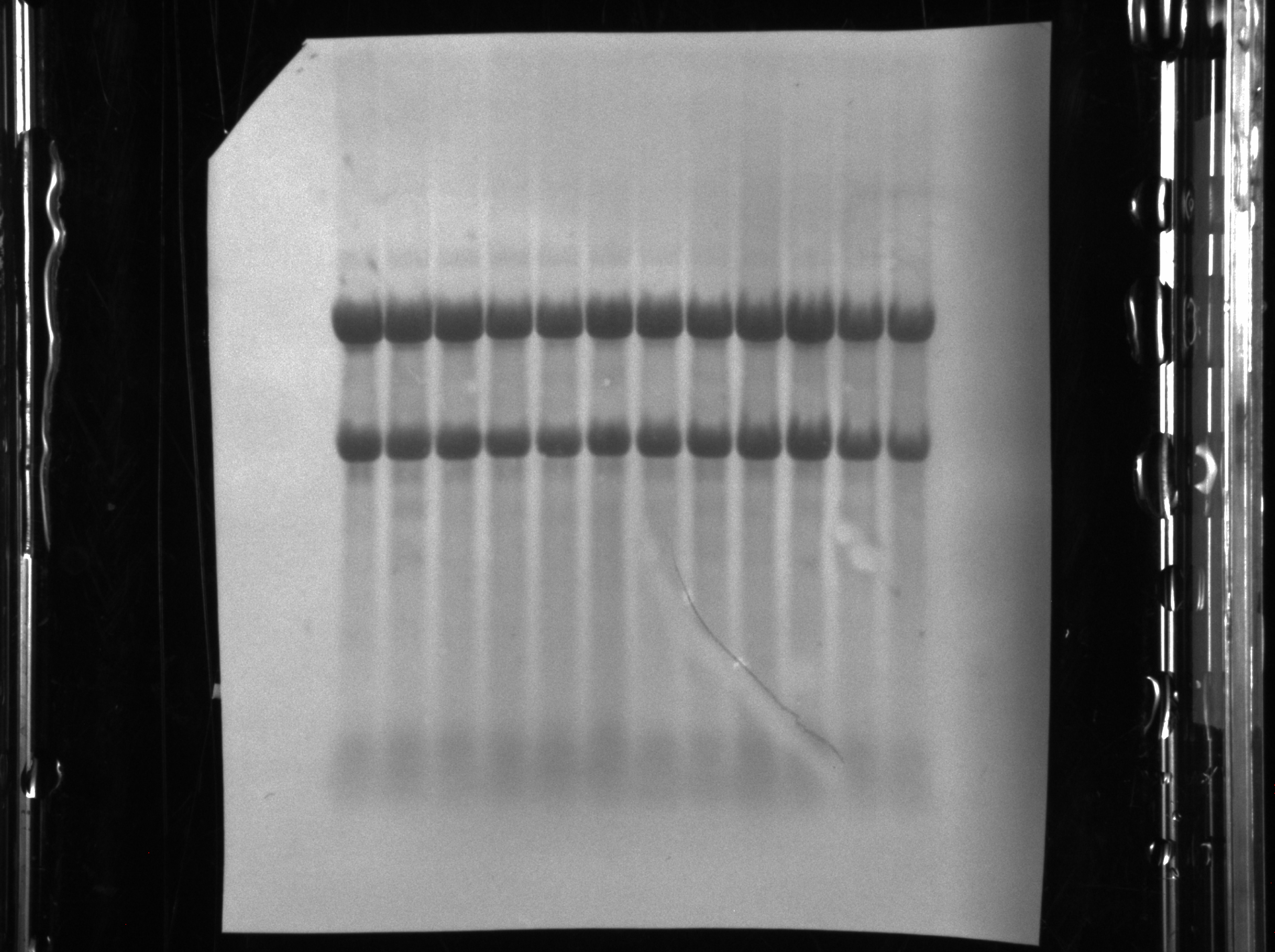

Supplement: Figure 5—source data 6. [file elife-90425-fig5-data6.zip › Figure 5 source data 6/5G_rRNA_methylene-blue_RNA-blot_uncropped.tif]

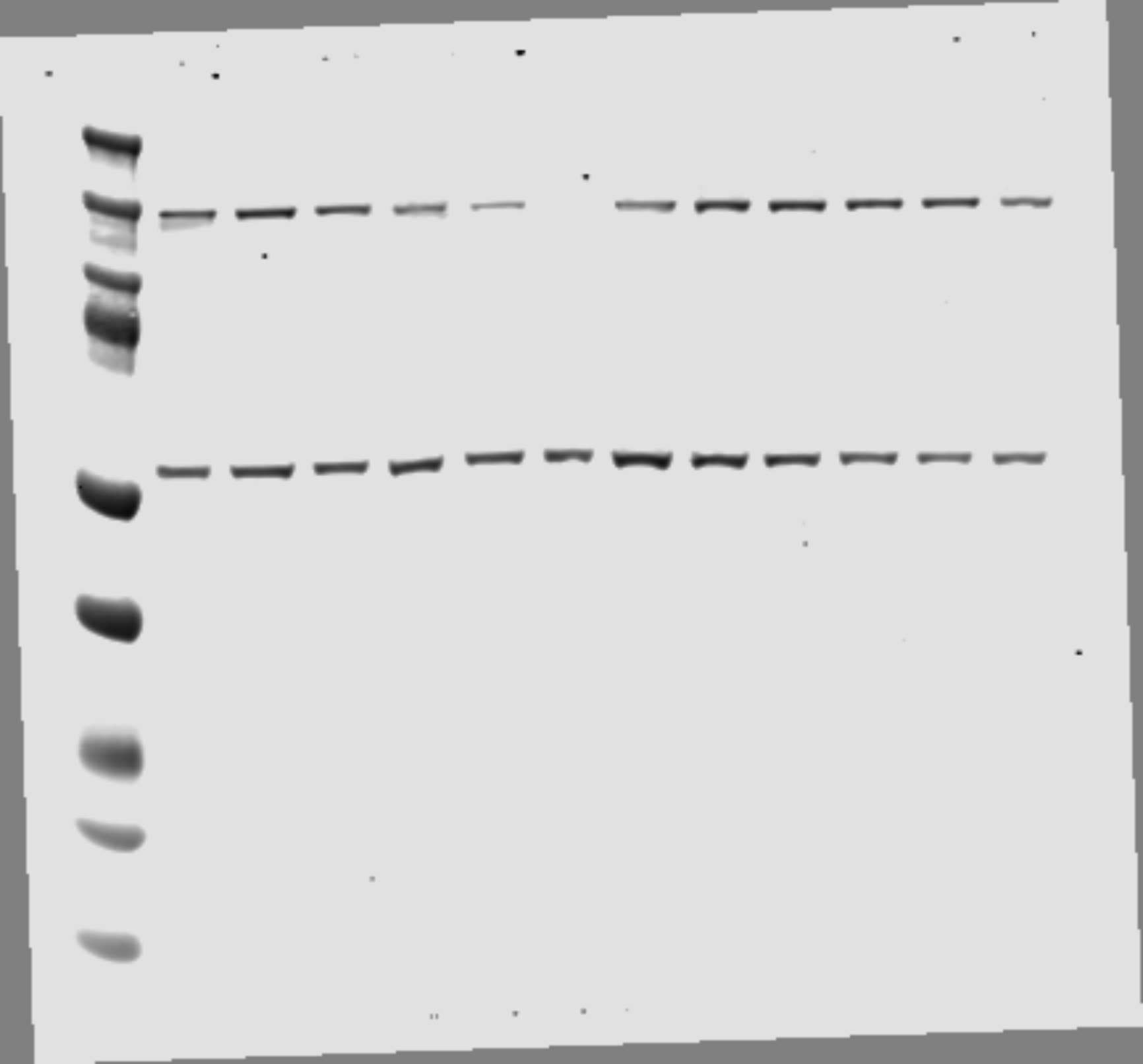

Supplement: Figure 5—source data 7. [file elife-90425-fig5-data7.zip › Figure 5 source data 7/5G_Swi4_Hxk2_Protein-blot_uncropped.tif]

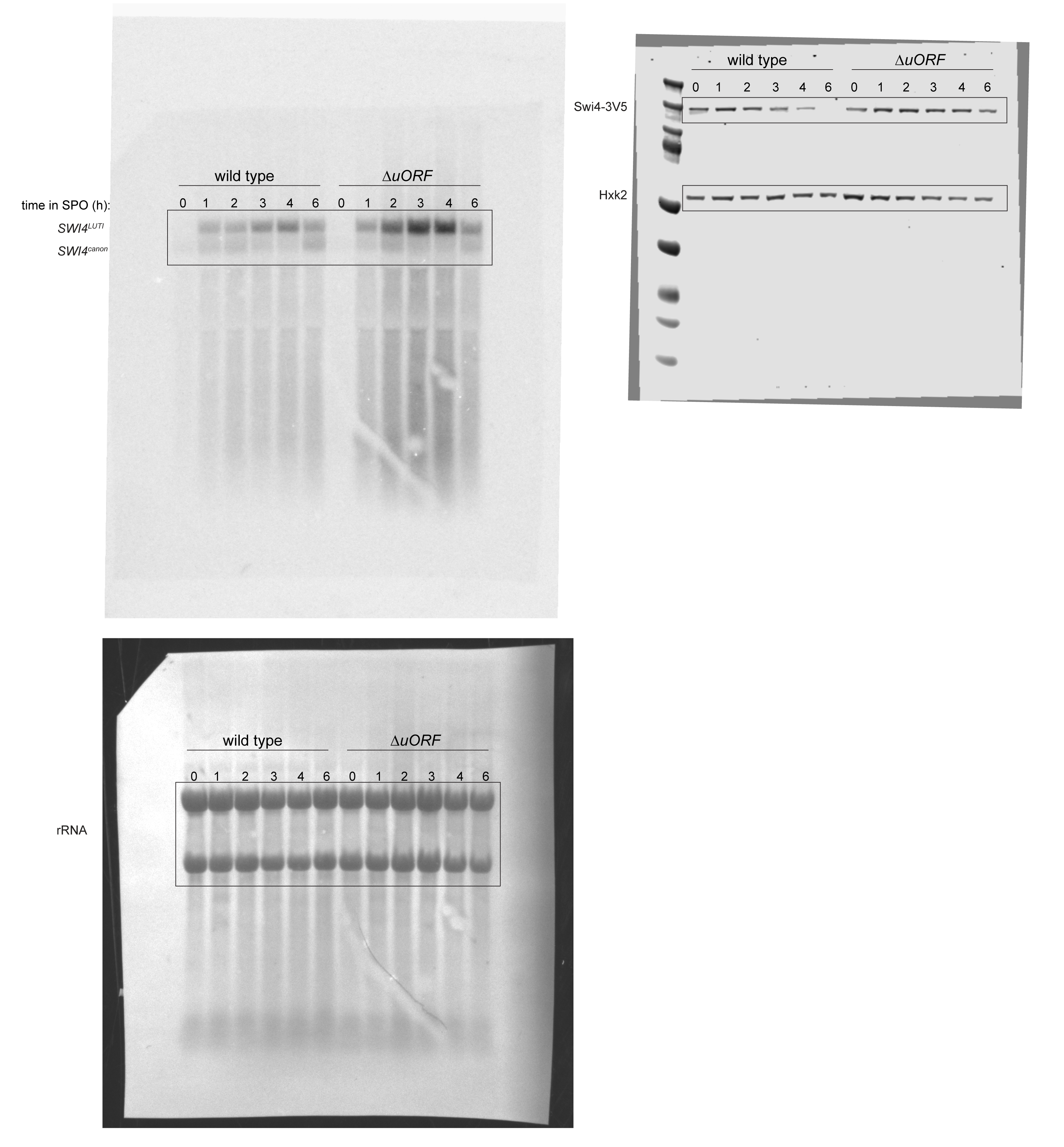

Supplement: Figure 5—source data 8. [file elife-90425-fig5-data8.zip › Figure 5 source data 8/5G_uncropped_with-labels.tif]

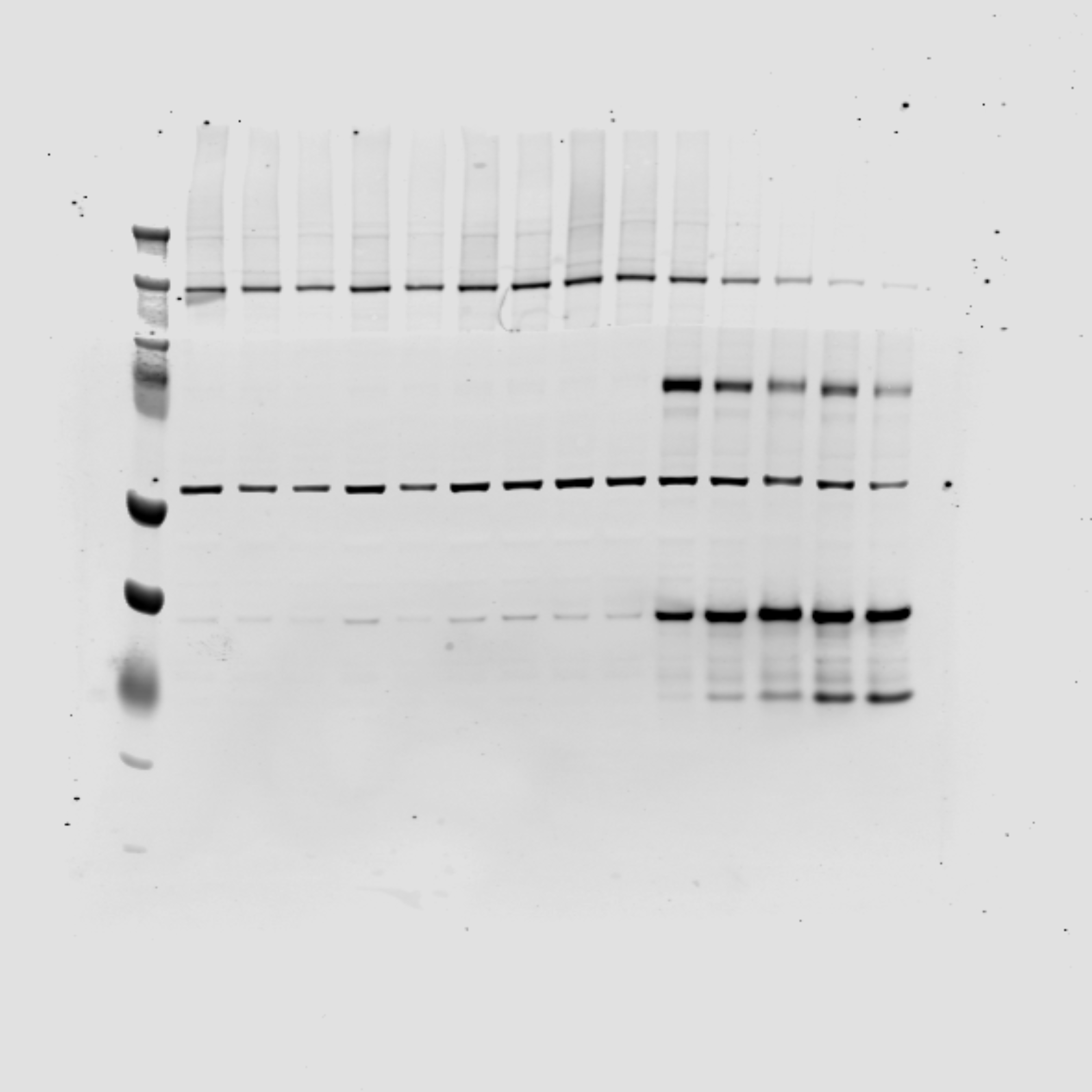

Supplement: Figure 5—source data 9. [file elife-90425-fig5-data9.zip › Figure 5 source data 9/5I_Swi4_Hxk2_uncropped.tif]

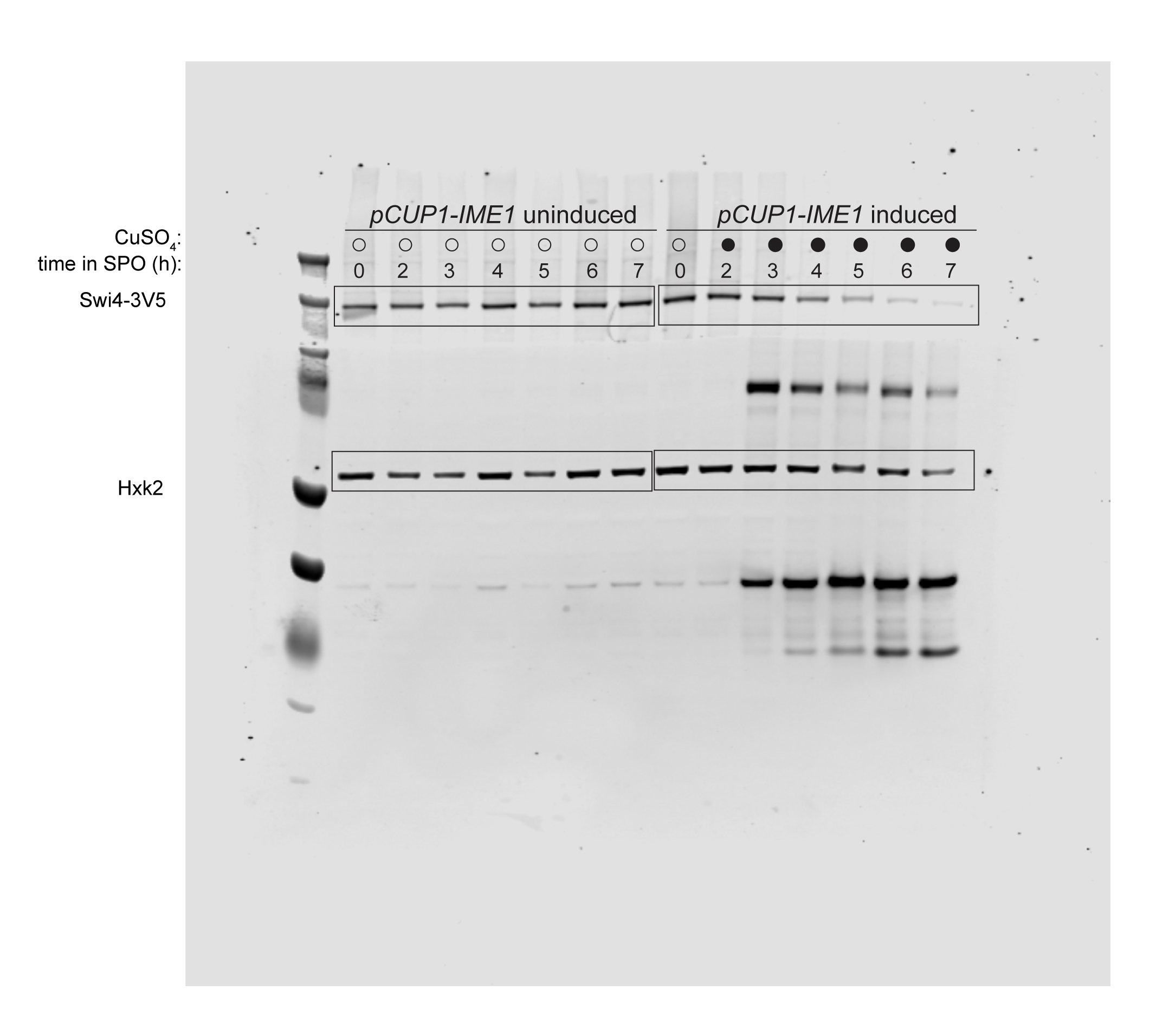

Supplement: Figure 5—source data 10. [file elife-90425-fig5-data10.zip › Figure 5 source data 10/5I_uncropped_with-labels.tif]

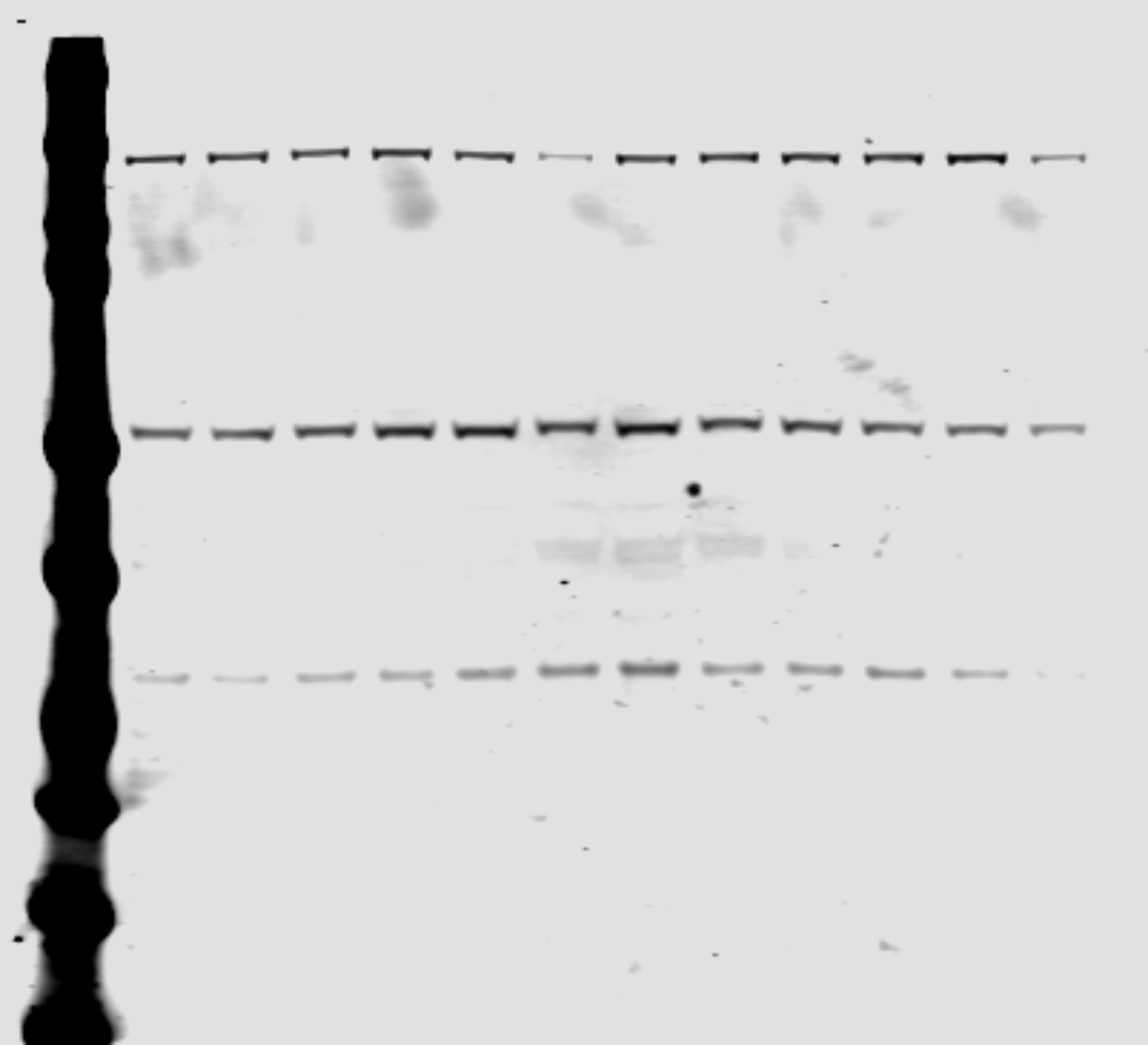

Supplement: Figure 6—figure supplement 2—source data 1. [file elife-90425-fig6-figsupp2-data1.zip › Figure 6-figure supplement 2 source data 1/Figure 6-figure supplement 2_Swi4_Hxk2_wild-type_ΓêåLUTI_uncropped.tif]

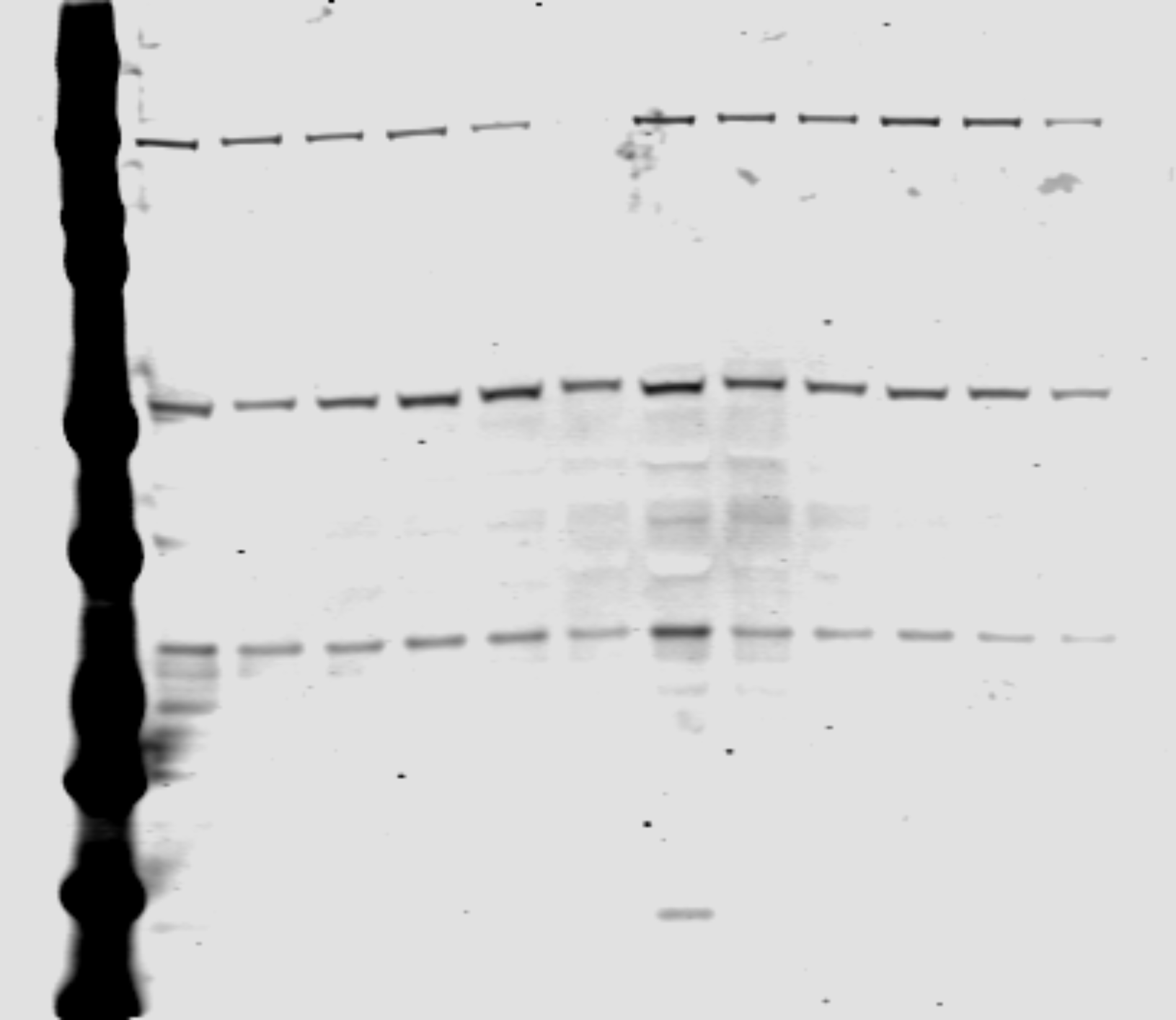

Supplement: Figure 6—figure supplement 2—source data 2. [file elife-90425-fig6-figsupp2-data2.zip › Figure 6-figure supplement 2 source data 2/Figure 6-figure supplement 2_Swi4_Hxk2_Whi5-AA_ΓêåLUTI;Whi5-AA_uncropped.tif]

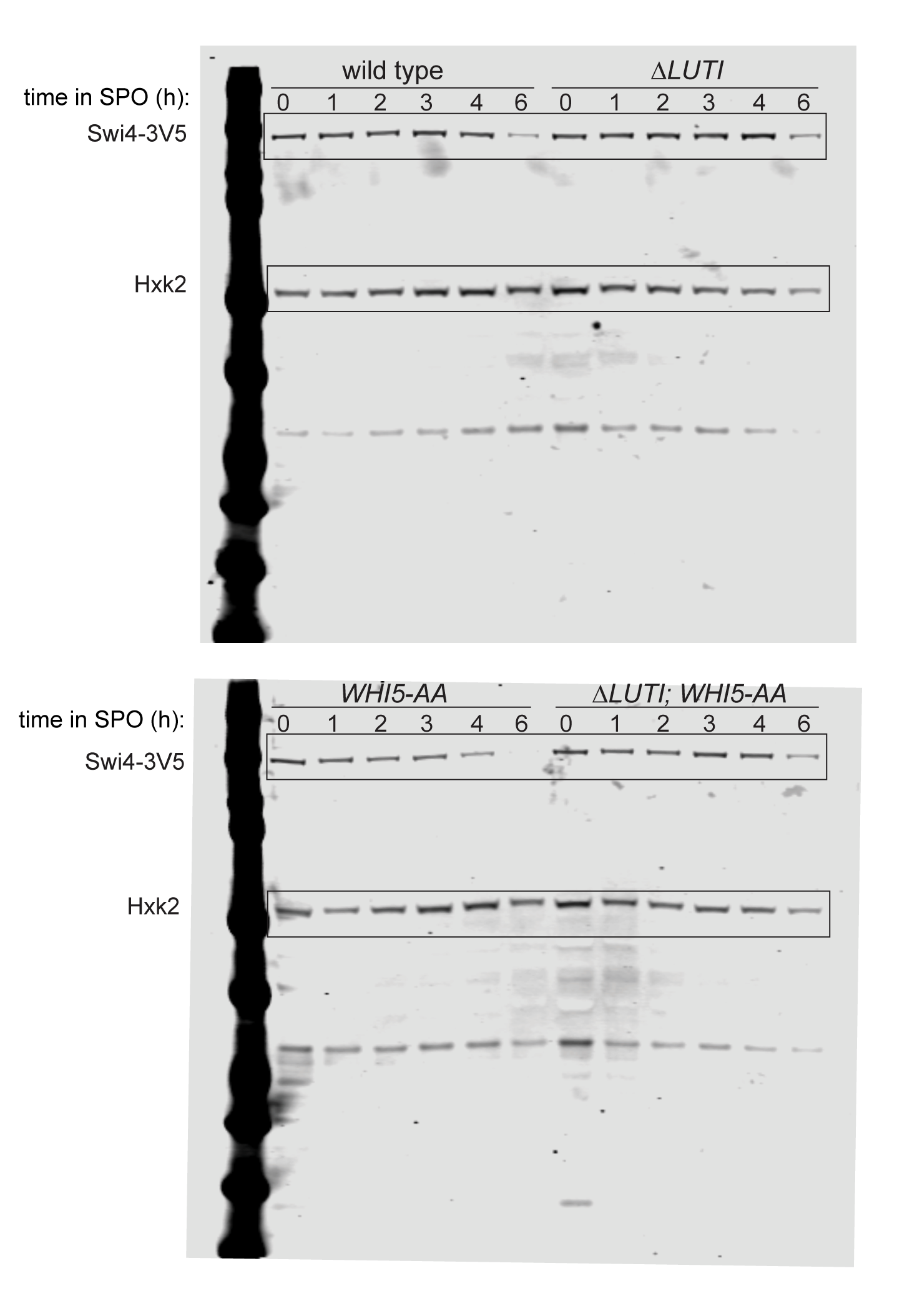

Supplement: Figure 6—figure supplement 2—source data 3. [file elife-90425-fig6-figsupp2-data3.zip › Figure 6-figure supplement 2 source data 3/Figure 6-figure supplement 2_uncropped_with-labels.tif]
