# Supplementary material for: Control of meiotic entry by dual inhibition of a key mitotic transcription factor
Source: eLife. 2024 Feb 27;12:RP90425. doi: 10.7554/eLife.90425 (PMC10939502; doi:10.7554/eLife.90425)
Supplement: Supplementary file 2. — 200 cells counted per strain. Second table is sporulation efficiency of cells at 24 hr in SPO media for the following genotypes: wild type (UB22199), pATG8-CLN1 (UB32820), pATG8-CLN2 (UB25959), pCUP-GFP-IME1 (UB34641), pCUP1-GFP-IME1; pATG8-CLN2 (UB35057), PUS1-αGFP (UB35593), PUS1-αGFP; pATG8-CLN2 (UB35982), UME6-αGFP (UB35300), and UME6-αGFP; pATG8-CLN2 (UB35177). [file elife-90425-supp2.docx]

| **Genotype** | **Sporulation Efficiency (%)** | **SD** | **n** | **two-tailed t-test vs. wild type** |
| --- | --- | --- | --- | --- |
| Rec8-GFP | 94.4 | 2.323 | 3 | ns, p = 0.1434 |
| *pATG8-SWI4;* Rec8-GFP | 73.0 | 2.598 | 3 | ***, p = 0.0002 |

See Figure 4A.

| **Genotype** | **Sporulation Efficiency (%)** | **n** | **two-tailed t test vs control** |
| --- | --- | --- | --- |
| wild type | 97.3 |  | N/A |
| *pATG8-CLN1* | 71.5 | 2 | *, p = 0.0171 |
| *pATG8-CLN2* | 8.5 | 4 | ****, p < 0.0001 |
| *pCUP-IME1; pATG8-CLN2* | 7 | 2 | **, p = 0.0052 |
| *PUS1-αGFP; pATG8-CLN2* | 7 | 2 | *, p = 0.0229 |
| *UME6-αGFP; pATG8-CLN2* | 91 | 2 | ns, p = 0.2943 |
