# Supplementary material for: Control of meiotic entry by dual inhibition of a key mitotic transcription factor
Source: eLife. 2024 Feb 27;12:RP90425. doi: 10.7554/eLife.90425 (PMC10939502; doi:10.7554/eLife.90425)
Supplement: Supplementary file 4. [file elife-90425-supp4.docx]

**Genotypes for the strains used in this study**

| **Strain** | **Genotype** |
| --- | --- |
| *SK1*  *wild-type* | *ho::LYS2 lys2 ura3 leu2::hisG his3::hisG trp1::hisG* |
| 14273 | *MAT****a****/alpha SWI4-3V5::KanMX/SWI4-3V5::KanMX pCUP-IME1::NAT/pCUP-IME1::NAT pCUP-IME4::NAT/pCUP-IME4::NAT* |
| 15411 | *MAT****a****/alpha irt1:cup1::Hphmx/irt1:cup1::Hphmx ime4::cup1::NAT/ime4::cup1::NAT HisMX::Δ(-1000 to -800)-SWI4-3V5::KanMX/HisMX::Δ(-1000 to -800)-SWI4-3V5::KanMX* |
| 21386 | *MAT****a****/alpha swi4::KanMX/swi4::KanMX leu2::LEU2::pSWI4(-1200 to -1)-SWI4-3V5-3'UTR/leu2::LEU2::pSWI4(-1200 to -1)-SWI4-3V5-3'UTR ura3::CA-URA3/ura3::CA-URA3 trp1::TRP1/trp1::TRP1* |
| 22199 | *MAT****a****/alpha HTB1-mCherry-HISMX6/HTB1-mCherry-HISMX6 ime1-N-sfGFP/ime1-N-sfGFP swi4::KanMX/swi4::KanMX leu2::LEU2::pSWI4(-1200 to -1)-SWI4-3V5-3'UTR/leu2::LEU2::pSWI4(-1200 to -1)-SWI4-3V5-3'UTR ura3::CA-URA3/ura3::CA-URA3 trp1::TRP1/ trp1::TRP1* |
| 22226 | *MAT****a****/alpha HTB1-mCherry-HISMX6/HTB1-mCherry-HISMX6 ime1-N-sfGFP/ime1-N-sfGFP leu2::pATG8-SWI4-3V5::LEU2/leu2::pATG8-SWI4-3V5::LEU2 ura3::CA-URA3/ura3::CA-URA3 trp1::TRP1/trp1::TRP1* |
| 23012 | *MAT****a****/alpha HTB1-mCherry-HISMX6/HTB1-mCherry-HISMX6 ime1-N-sfGFP/ime1-N-sfGFP swi4::KanMX/swi4::KanMX leu2::LEU2::pSWI4LUTI(-1200 to -934)Δ-SWI4-3V5-SWI4_3'UTR/leu2::LEU2::pSWI4LUTI(-1200 to -934)Δ-SWI4-3V5-SWI4_3'UTR ura3::CA-URA3/ura3::CA-URA3 trp1::TRP1/trp1::TRP1* |
| 23636 | *MAT****a****/alpha* leu2::LEU2::pSWI4(-1200 to -1)∆7AUG-uORF-SWI4-3V5-3'UTR/leu2::LEU2::pSWI4(-1200 to -1)∆7AUG-uORF-SWI4-3V5-3'UTR swi4::KanMX/swi4::KanMX ura3::CA-URA3/ura3::CA-URA3 trp1::TRP1/trp1::TRP1 |
| 25428 | *MAT****a****/alpha* whi5::WHI5-mcherry-linker-FRB::KanMX/whi5::WHI5-mcherry-linker-FRB::KanMX RPL13a-2xFKBP12::TRP1/RPL13a-2xFKBP12::TRP1 fpr1::KanMX/fpr1::KanMX tor1-1::HIS3/tor1-1::HIS3 swi4::KanMX/swi4::KanMX leu2::LEU2::pSWI4LUTI(-1200 to -934)Δ-SWI4-3V5-SWI4_3'UTR/leu2::LEU2::pSWI4LUTI(-1200 to -934)Δ-SWI4-3V5-SWI4_3'UTR ura3::CA-URA3/ura3::CA-URA3 |
| 25431 | *MAT****a****/alpha* whi5::WHI5-mcherry-linker-FRB::KanMX/whi5::WHI5-mcherry-linker-FRB::KanMX RPL13a-2xFKBP12::TRP1/RPL13a-2xFKBP12::TRP1 fpr1::KanMX/fpr1::KanMX tor1-1::HIS3/tor1-1::HIS3 swi4::KanMX/swi4::KanMX leu2::LEU2::pSWI4(-1200 to -1)-SWI4-3V5-3'UTR/leu2::LEU2::pSWI4(-1200 to -1)-SWI4-3V5-3'UTR ura3::CA-URA3/ura3::CA-URA3 |
| 25959 | *MAT****a****/alpha his3::HIS3::pATG8-CLN2-3V5/his3::HIS3::pATG8-CLN2-3V5 HTB1-mCherry-HISMX6/HTB1-mCherry-HISMX6 ime1-N-sfGFP/ime1-N-sfGFP ura3::CA-URA3/ura3::CA-URA3 trp1::TRP1/trp1::TRP1* |
| 25982 | *MAT****a****/alpha his3::HIS3::pATG8-CLN2-3V5/his3::HIS3::pATG8-CLN2-3V5 Pus1-VH16::URA3/Pus1-VH16::URA3 HTB1-mCherry-HISMX6/HTB1-mCherry-HISMX6 ime1-N-sfGFP/ime1-N-sfGFP swi4::KanMX/swi4::KanMX leu2::LEU2::pSWI4(-1200 to -1)-SWI4-3V5-3'UTR/leu2::LEU2::pSWI4(-1200 to -1)-SWI4-3V5-3'UTR trp1::TRP1/trp1::TRP1* |
| 26874 | *MAT****a****/alpha* whi5::WHI5-mcherry-linker-FRB::KanMX/whi5::WHI5-mcherry-linker-FRB::KanMX fpr1::KanMX/fpr1::KanMX tor1-1::HIS3/tor1-1::HIS3 swi4::KanMX/swi4::KanMX leu2::LEU2::pSWI4LUTI(-1200 to -934)Δ-SWI4-3V5-SWI4_3'UTR/leu2::LEU2::pSWI4LUTI(-1200 to -934)Δ-SWI4-3V5-SWI4_3'UTR ura3::CA-URA3/ura3::CA-URA3 trp1::TRP1/trp1::TRP1 |
| 27083 | *MAT****a****/alpha* whi5::WHI5-mcherry-linker-FRB::KanMX/whi5::WHI5-mcherry-linker-FRB::KanMX fpr1::KanMX/fpr1::KanMX tor1-1::HIS3/tor1-1::HIS3 swi4::KanMX/swi4::KanMX leu2::LEU2::pSWI4(-1200 to -1)-SWI4-3V5-3'UTR/leu2::LEU2::pSWI4(-1200 to -1)-SWI4-3V5-3'UTR ura3::CA-URA3/ura3::CA-URA3 trp1::TRP1/trp1::TRP1 |
| 29326 | *MAT****a****/alpha CLN1-3V5::HYG/CLN1-3V5::HYG HTB1-mCherry-HISMX6/HTB1-mCherry-HISMX6 ime1-N-sfGFP/ime1-N-sfGFP swi4::KanMX/swi4::KanMX leu2::LEU2::pSWI4(-1200 to -1)-SWI4-3V5-3'UTR/leu2::LEU2::pSWI4(-1200 to -1)-SWI4-3V5-3'UTR ura3::CA-URA3/ura3::CA-URA3 trp1::TRP1/trp1::TRP1* |
| 29328 | *MAT****a****/alpha CLN1-3V5::HYG/CLN1-3V5::HYG HTB1-mCherry-HISMX6/HTB1-mCherry-HISMX6 ime1-N-sfGFP/ime1-N-sfGFP leu2::pATG8-SWI4-3V5::LEU2/leu2::pATG8-SWI4-3V5::LEU2 ura3::CA-URA3/ura3::CA-URA3 trp1::TRP1/trp1::TRP1* |
| 29330 | *MAT****a****/alpha CLN2-3v5::KAN/CLN2-3v5::KAN HTB1-mCherry-HISMX6/HTB1-mCherry-HISMX6 ime1-N-sfGFP/ime1-N-sfGFP swi4::KanMX/swi4::KanMX*  *leu2::LEU2::pSWI4(-1200 to -1)-SWI4-3V5-3'UTR/leu2::LEU2::pSWI4(-1200 to -1)-SWI4-3V5-3'UTR ura3::CA-URA3/ura3::CA-URA3 trp1::TRP1/trp1::TRP1* |
| 29332 | *MAT****a****/alpha CLN2-3v5::KAN/CLN2-3v5::KAN HTB1-mCherry-HISMX6/HTB1-mCherry-HISMX6 ime1-N-sfGFP/ime1-N-sfGFP leu2::pATG8-SWI4-3V5::LEU2/leu2::pATG8-SWI4-3V5::LEU2 ura3::CA-URA3/ura3::CA-URA3*  *trp1::TRP1/trp1::TRP1* |
| 31378 | *MAT****a****/alpha swi4::KanMX/swi4::KanMX leu2::LEU2::pSWI4(-1200 to -1)-SWI4-mCherry::HIS3/leu2::LEU2::pSWI4(-1200 to -1)-SWI4-mCherry::HIS3 ime1-N-sfGFP/ime1-N-sfGFP ura3::CA-URA3/ura3::CA-URA3 trp1::TRP1/trp1::TRP1* |
| 31381 | *MAT****a****/alpha leu2::LEU2::pATG8-SWI4-mCherry::HIS3/leu2::LEU2::pATG8-SWI4-mCherry::HIS3 ime1-N-sfGFP/ime1-N-sfGFP ura3::CA-URA3/ura3::CA-URA3 trp1::TRP1/trp1::TRP1* |
| 32820 | *MAT****a****/alpha trp1::TRP1::pATG8-CLN1-3V5/trp1::TRP1::pATG8-CLN1-3V5*  *HTB1-mCherry-HISMX6/HTB1-mCherry-HISMX6 ime1-N-sfGFP/ime1-N-sfGFP ura3::CA-URA3/ura3::CA-URA3* |
| 32085 | *MAT****a****/alpha swi4::KanMX/swi4::KanMX leu2::LEU2::pSWI4(-1200 to -1)-SWI4-3V5-3'UTR/leu2::LEU2::pSWI4(-1200 to -1)-SWI4-3V5-3'UTR HTB1-mCherry-HISMX6/HTB1-mCherry-HISMX6 REC8-GFP-URA3/REC8-GFP-URA3 trp1::TRP1/trp1::TRP1* |
| 32089 | *MAT****a****/alpha leu2::LEU2::pATG8-SWI4-3V5/leu2::LEU2::pATG8-SWI4-3V5 HTB1-mCherry-HISMX6/HTB1-mCherry-HISMX6 REC8-GFP-URA3/REC8-GFP-URA3 trp1::TRP1/trp1::TRP1* |
| 34165 | *MAT****a****/alpha cln2∆::NatMX/cln2∆::NatMX leu2::pATG8-SWI4-3V5::LEU2/leu2::pATG8-SWI4-3V5::LEU2 REC8-GFP-URA3/REC8-GFP-URA3 HTB1-mCherry-HISMX6/HTB1-mCherry-HISMX6 trp1::TRP1/trp1::TRP1* |
| 34536 | *MAT****a****/alpha cln1∆::hyg/cln1∆::hyg leu2::LEU2::pATG8-SWI4-3V5/leu2::LEU2::pATG8-SWI4-3V5 HTB1-mCherry-HISMX6/HTB1-mCherry-HISMX6 REC8-GFP-URA3 /REC8-GFP-URA3 trp1::TRP1/trp1::TRP1* |
| 34641 | *MAT****a****/alpha HTB1-mCherry-HISMX6/HTB1-mCherry-HISMX6 KanMX:pCUP-ime1-N-sfGFP/KanMX:pCUP-ime1-N-sfGFP swi4::KanMX/swi4::KanMX leu2::LEU2::pSWI4(-1200 to -1)-SWI4-3V5-3'UTR/leu2::LEU2::pSWI4(-1200 to -1)-SWI4-3V5-3'UTR ura3::CA-URA3/ura3::CA-URA3 trp1::TRP1/trp1::TRP1* |
| 35057 | *MAT****a****/alpha HTB1-mCherry-HISMX6/HTB1-mCherry-HISMX6 KanMX:pCUP-ime1-N-sfGFP/KanMX:pCUP-ime1-N-sfGFP swi4::KanMX/swi4::KanMX leu2::LEU2::pSWI4(-1200 to -1)-SWI4-3V5-3'UTR/leu2::LEU2::pSWI4(-1200 to -1)-SWI4-3V5-3'UTR his3::HIS3::pATG8-CLN2-3V5/his3::HIS3::pATG8-CLN2-3V5 ura3::CA-URA3/ura3::CA-URA3 trp1::TRP1/trp1::TRP1* |
| 35106 | *MAT****a****/alpha his3::HIS3::pATG8-CLN2-3V5/his3::HIS3::pATG8-CLN2-3V5 HTB1-mCherry-HISMX6/HTB1-mCherry-HISMX6 ime1-N-sfGFP/ime1-N-sfGFP swi4::KanMX/swi4::KanMX leu2::LEU2::pSWI4(-1200 to -1)-SWI4-3V5-3'UTR/leu2::LEU2::pSWI4(-1200 to -1)-SWI4-3V5-3'UTR ura3::CA-URA3/ura3::CA-URA3 trp1::TRP1/trp1::TRP1* |
| 35177 | *MAT****a****/alpha his3::HIS3::pATG8-CLN2-3V5/his3::HIS3::pATG8-CLN2-3V5*  *HTB1-mCherry-HISMX6/HTB1-mCherry-HISMX6 ime1-N-sfGFP/ime1-N-sfGFP swi4::KanMX/swi4::KanMX leu2::LEU2::pSWI4(-1200 to -1)-SWI4-3V5-3'UTR/leu2::LEU2::pSWI4(-1200 to -1)-SWI4-3V5-3'UTR UME6-VH16::URA3/UME6-VH16::URA3 trp1::TRP1/trp1::TRP1* |
| 35246 | *MAT****a****/alpha ura3::CA-URA3/ura3::CA-URA3 trp1::TRP1/trp1::TRP1* |
| 35300 | *MAT****a****/alpha HTB1-mCherry-HISMX6/HTB1-mCherry-HISMX6 ime1-N-sfGFP/ime1-N-sfGFP swi4::KanMX/swi4::KanMX leu2::LEU2::pSWI4(-1200 to -1)-SWI4-3V5-3'UTR/leu2::LEU2::pSWI4(-1200 to -1)-SWI4-3V5-3'UTR UME6-VH16::URA3/UME6-VH16::URA3 trp1::TRP1/trp1::TRP1* |
| 35593 | *MAT****a****/alpha Pus1-VH16::URA3/Pus1-VH16::URA3 HTB1-mCherry-HISMX6/HTB1-mCherry-HISMX6 ime1-N-sfGFP/ime1-N-sfGFP swi4::KanMX/swi4::KanMX leu2::LEU2::pSWI4(-1200 to -1)-SWI4-3V5-3'UTR/leu2::LEU2::pSWI4(-1200 to -1)-SWI4-3V5-3'UTR trp1::TRP1/trp1::TRP1* |
| 35595 | *MATa/alpha cln2∆::NatMX/cln2∆::NatMX swi4::KanMX/swi4::KanMX leu2::LEU2::pSWI4(-1200 to -1)-SWI4-3V5-3'UTR/leu2::LEU2::pSWI4(-1200 to -1)-SWI4-3V5-3'UTR REC8-GFP-URA3/REC8-GFP-URA3 HTB1-mCherry-HISMX6/HTB1-mCherry-HISMX6 trp1::TRP1/trp1::TRP1* |
| 35597 | *MATa/alpha cln1∆::hyg/cln1∆::hyg swi4::KanMX/swi4::KanMX leu2::LEU2::pSWI4(-1200 to -1)-SWI4-3V5-3'UTR/leu2::LEU2::pSWI4(-1200 to -1)-SWI4-3V5-3'UTR HTB1-mCherry-HISMX6/HTB1-mCherry-HISMX6 REC8-GFP-URA3/REC8-GFP-URA3 trp1::TRP1/trp1::TRP1* |
| 35985 | *MAT****a****/alpha whi5::WHI5-mcherry-linker-FRB::KanMX/whi5::WHI5-mcherry-linker-FRB::KanMX RPL13a-2xFKBP12::TRP1/RPL13a-2xFKBP12::TRP1 fpr1::KanMX/fpr1::KanMX tor1-1::HIS3/tor1-1::HIS3 HTB1-mCherry-HISMX6/HTB1-mCherry-HISMX6 REC8-GFP-URA3/REC8-GFP-URA3 swi4::KanMX/swi4::KanMX leu2::LEU2::pSWI4L UTI(-1200 to -934)Δ-SWI4-3V5-SWI4_3'UTR/leu2::LEU2::pSWI4L UTI(-1200 to -934)Δ-SWI4-3V5-SWI4_3'UTR trp1::TRP1/trp1::TRP1* |
| 35987 | *MAT****a****/alpha whi5::WHI5-mcherry-linker-FRB::KanMX/whi5::WHI5-mcherry-linker-FRB::KanMX tor1-1::HIS3/tor1-1::HIS3 fpr1::KanMX/fpr1::KanMX HTB1-mCherry-HISMX6/HTB1-mCherry-HISMX6 REC8-GFP-URA3/REC8-GFP-URA3 swi4::KanMX/swi4::KanMX leu2::LEU2::pSWI4(-1200 to -1)-SWI4-3V5-3'UTR/leu2::LEU2::pSWI4(-1200 to -1)-SWI4-3V5-3'UTR trp1::TRP1/trp1::TRP1* |
| 35989 | *MAT****a****/alpha swi4::KanMX/swi4::KanMX leu2::LEU2::pSWI4LUTI(-1200 to -934)Δ-SWI4-3V5-SWI4_3'UTR/leu2::LEU2::pSWI4LUTI(-1200 to -934)Δ-SWI4-3V5-SWI4_3'UTR whi5::WHI5-mcherry-linker-FRB::KanMX/whi5::WHI5-mcherry-linker-FRB::KanMX fpr1::KanMX/fpr1::KanMX tor1-1::HIS3/tor1-1::HIS3 HTB1-mCherry-HISMX6/HTB1-mCherry-HISMX6 REC8-GFP-URA3 /REC8-GFP-URA3 trp1::TRP1/trp1::TRP1* |
| 35991 | *MAT****a****/alpha whi5::WHI5-mcherry-linker-FRB::KanMX whi5::WHI5-mcherry-linker-FRB::KanMX RPL13a-2xFKBP12::TRP1/RPL13a-2xFKBP12::TRP1 fpr1::KanMX/fpr1::KanMX tor1-1::HIS3/tor1-1::HIS3 HTB1-mCherry-HISMX6/HTB1-mCherry-HISMX6 REC8-GFP-URA3/REC8-GFP-URA3 swi4::KanMX/swi4::KanMX leu2::LEU2::pSWI4(-1200 to -1)-SWI4-3V5-3'UTR/leu2::LEU2::pSWI4(-1200 to -1)-SWI4-3V5-3'UTR* |
