# Supplementary material for: Control of meiotic entry by dual inhibition of a key mitotic transcription factor
Source: eLife. 2024 Feb 27;12:RP90425. doi: 10.7554/eLife.90425 (PMC10939502; doi:10.7554/eLife.90425)
Supplement: Supplementary file 5. [file elife-90425-supp5.docx]

**Plasmids used in this study**

| **Plasmid Number** | **Plasmid Info** |
| --- | --- |
| pUB595 | pFA6a-FRB-KanMX6 |
| pUB1585 | LEU2-pATG8-SWI4-linker-3V5 |
| pUB1587 | LEU2-pSWI4(-1200 to -1)-SWI4-3V5-3′UTR |
| pUB1588 | LEU2-pSWI4(-1200 to -934)Δ-SWI4-3V5-3′UTR (LUTI∆) |
| pUB1734 | LEU2-pSWI4(ATG>ATC mutant)-SWI4-3V5-3’UTR (uORF∆) |
| pUB1899 | HIS3-pATG8-CLN2-linker-3V5 |
| pUB2144 | TRP1-pATG8-CLN1-linker-3V5 |
