# Supplementary material for: Control of meiotic entry by dual inhibition of a key mitotic transcription factor
Source: eLife. 2024 Feb 27;12:RP90425. doi: 10.7554/eLife.90425 (PMC10939502; doi:10.7554/eLife.90425)
Supplement: Supplementary file 6. [file elife-90425-supp6.docx]

**Primers used for quantitative PCR and RNA blotting**

| **Primer Name** | **Sequence from 5′ to 3′** |
| --- | --- |
| 6852_CLN2_F | TCGTGTTACGGGACCAAGCC |
| 6853_CLN2_R | TACGTGCCCTTGGGTTGGGA |
| 6887_CLN1_F | ACGTCTCCATCCCCACAGGT |
| 6888_CLN1_R | CGGACCCGCCGCAATAATGA |
| 3301_PFY1_F | ACGGTAGACATGATGCTGAGG |
| 3302_PFY1_R | ACGGTTGGTGGATAATGAGC |
| 2081_IME1_F | TCACCACCGCCATCACTACA |
| 2082_IME1_R | TGAAGGAGTAAGCCGCAGCA |
| 6854_CDC21_F | TTGGCCGGTGATACAGACGC |
| 6855_CDC21_R | ACGGGCCCCAGATCTCCTAC |
| 6858_RNR1_F | ACCCTAGCGGCCAGAATTGC |
| 6859_RNR1_R | CATGGGAGCGGGCTTACCAG |
| 2598_ACT1_F | GTACCACCATGTTCCCAGGTATT |
| 2599_ACT1_R | AGATGGACCACTTTCGTCGT |
| 5429_SWI4LUTI_F | ACAAGGACTAAGAAGCACGTCA |
| 5430_SWI4LUTI_R | ACCAATGCTAAAGGATGGCA |
| 5918_3V5_probe_F | CTAGTGGATCCAGGTAAACCTAT |
| 2921_3V5_probe_R | TAATACGACTCACTATAGGCCAGTCCTAATAGAGGATTAGG |
