# Supplementary material for: Control of meiotic entry by dual inhibition of a key mitotic transcription factor
Source: eLife. 2024 Feb 27;12:RP90425. doi: 10.7554/eLife.90425 (PMC10939502; doi:10.7554/eLife.90425)
Supplement: Supplementary file 7. [file elife-90425-supp7.docx]

**Image acquisition settings**

| **Genotype** | **Figure** | **GFP** | **RFP** | **DAPI** | **POL** |
| --- | --- | --- | --- | --- | --- |
| GFP-Ime1; Htb1-mCherry | 1F, 2A-2B, 4B, 4E, 4H, 4K | 50% T, 0.1s  EX: 475/28  EM: 523/36 | 32% T, 0.05s  EX: 575/25  EM: 632/60 | N/A | 32% T, 0.1s |
| GFP-Ime1; Swi4-mCherry | 2C-2E | 50% T, 0.1s  EX: 475/28  EM: 523/36 | 50% T, 0.05s  EX: 575/25  EM: 632/60 | 32% T,  0.05s  EX: 390/18  EM: 435/48 | 32% t, 0.1s |
| GFP-Ime1; Whi5-mCherry | 6B,  6-sup1 | 50% T, 0.1s  EX: 475/28  EM: 523/36 | 100% T, 0.08s  EX: 575/25  EM: 632/60 | 32% T,  0.05s  EX: 390/18  EM: 435/48 | 32% T, 0.1s |
| Rec8-GFP;  Htb1-mCherry | 2H-2I | 10% T, 0.025s  EX: 475/28  EM: 523/36 | 10% T, 0.025s  EX: 575/25  EM: 632/60 | N/A | 32% t, 0.1s |
